# How trait confidence and communication shape dyadic decision outcomes and confidence matching

**DOI:** 10.1186/s41235-026-00705-1

**Published:** 2026-01-31

**Authors:** Matthew D. Blanchard, Eugene Aidman, Lazar Stankov, Sabina Kleitman

**Affiliations:** 1 School of Psychology, The University of Sydney, A18, Room 440, Sydney, NSW 2006 Australia; 2 School of Biomedical Sciences and Pharmacy, University of Newcastle, Newcastle, NSW 2300 Australia; 3 Human & Decision Sciences Division, Defence Science and Technology Group, Third Avenue, Edinburgh, SA 5111 Australia

**Keywords:** Confidence, Metacognition, Confidence matching, Dyads, Decision making

## Abstract

**Abstract:**

When individuals collaborate, they often rely on momentary estimates of their own and their partner’s confidence (decision confidence) to guide collective decisions and achieve their goals. Through interaction, these confidence estimates tend to align over time. This process is known as confidence matching. More stable, dispositional trait confidence is also emerging as a key factor shaping the dynamics and outcomes of collaborative action. We examined how trait confidence and type of communication impact the accuracy of dyadic decisions, decision confidence, and the dynamics of decision confidence, including decision-specific confidence matching.

In this study, 210 participants completed general knowledge tests individually and collaboratively, forming 105 dyads. The tests were completed under three communication conditions: isolated (no interaction), passive (viewing the partner’s response and numeric confidence rating), and active (verbal discussion). Participants assessed as high-trait or low-trait confidence were allocated to three types of dyads: low-trait (two low-trait members), mixed-trait (one low-trait and one high-trait member), or high-trait (two high-trait members) confidence dyads.

Statistically controlling for cognitive ability, trait confidence moderated decision accuracy and decision confidence gains: dyads with mixed-trait or high-trait confidence showed greater decision accuracy improvements in the active than the passive communication condition compared to their individual decisions. Whereas low-trait confidence dyads benefited equally from active and passive communication. Collaboration increased decision confidence overall, especially for high-trait confidence dyads under active communication. Decision-specific confidence matching occurred rapidly in both passive and active communication but predicted decision accuracy gains only in the passive condition where participants had limited social information.

Although active verbal communication led to the greatest overall decision accuracy, these gains were not driven by decision-specific confidence matching. Our findings highlight the critical role of trait confidence in shaping collaborative outcomes in dyads and extend previous research by showing that decision-specific confidence matching occurs naturally during verbal communication.

**Significance statement:**

When two people collaborate to make decisions, we often assume that “two heads are better than one.” However, the benefits of dyadic decision-making depend on how effectively group members share and interpret their confidence in judgments. Our study highlights trait confidence, an individual’s stable tendency to express confidence, as a critical yet often overlooked factor that shapes the success of dyadic decisions. We found that trait confidence moderates dyadic improvements in both decision accuracy and decision confidence. Importantly, the effectiveness of dyadic collaboration depends on the type of communication: verbal discussions maximized accuracy gains for dyads with high or mixed levels of trait confidence, whereas simpler, non-verbal exchanges were sufficient for low-trait confidence dyads. Additionally, we demonstrated that dyad members naturally align their levels of confidence (a process known as confidence matching) during verbal discussions*.* This extends prior research showing that the language used to express confidence becomes more similar over time and that confidence matching has previously been observed only under artificial, numeric rating contexts. These insights enhance our understanding of how individual differences in trait confidence and communication modes influence collaborative decisions, providing practical guidance for effectively structuring collaborative interactions and pairing partners in applied settings.

**Supplementary Information:**

The online version contains supplementary material available at 10.1186/s41235-026-00705-1.

## Collective decisions

How people share and integrate information in groups is critical for success (Hastie & Kameda, 2005). Collective decisions are typically characterised by uncertainty and choices among known options. For example, a driver and navigator must assess terrain features to determine the most efficient route, or a pair of intelligence analysts may evaluate conflicting reports to reach a consensus on the likelihood of enemy activity in a specified area. In such contexts, achieving a “two heads are better than one” effect depends not only on pooling information but also on communicating effectively, aligning confidence, and integrating perspectives into a joint decision. For dyads, simply following the majority is not viable, as ties are common. This raises an important question: how do dyads achieve a collective benefit?

Bahrami et al. (2010) provided an important extension on classic small group research (e.g., Festinger, 1954; Hill, 1982; Hinsz, 1990; Sniezek & Henry, 1989; Tindale, 1989) by showing that dyads achieve a collective benefit by sharing and using each other’s confidence. Confidence reflects the moment-to-moment monitoring of one’s performance (referred to as decision confidence; Koriat, 2008). This strategy works because higher-confidence ratings often signal a higher probability of being correct (Koriat, 2008, 2012b; Stankov & Crawford, 1996; Yaniv, 1997). Over repeated trials, partners’ confidence ratings tend to shift toward each other, an alignment process called “confidence matching” (Bang et al., 2017). Confidence matching is useful because partners may differ in baseline levels of trait confidence, which is a stable, domain-general tendency for confidence judgments across different tasks and contexts (e.g., Johnson, 1939; Kleitman & Stankov, 2001). Without matching, a consistently higher confidence partner may dominate joint decisions, even when they are not more likely to be correct (Blanchard et al., 2020). Aligning confidence scales helps dyads identify, on each trial, whose judgment is more likely to be correct. Several studies have examined this process (e.g., Friedemann et al., 2024; Schneider et al., 2024), including Pescetelli and Yeung (2022) who linked confidence matching to decision accuracy gains under more naturalistic conditions.

Working in pairs or small groups can also boost confidence (Patalano & LeClair, 2011; Savadori et al., 2001; Sniezek & Henry, 1989; Zarnoth & Sniezek, 1997) and this rise can occur when decision accuracy does not improve (Blanchard et al., 2020; Heath & Gonzalez, 1995; Minson & Mueller, 2012; Schuldt et al., 2017). Two recent studies demonstrate that the size of this increase depends on the trait confidence levels of the dyad members, suggesting that trait confidence plays an important role in how decision confidence develops within dyads (Blanchard et al., 2020; Schuldt et al., 2017). Decision confidence captures situational judgments, while trait confidence reflects broader individual differences in metacognitive self-beliefs (Stankov et al., 2015). Because each confidence judgment carries both task-specific and person-specific variance, trait confidence may shape how decision confidence develops during collaboration.

The present study examined trait confidence as a moderator of how dyads share information and improve decisions. We tested whether baseline trait confidence influences the magnitude of changes in collective decision accuracy and decision confidence relative to individual decisions, and whether it moderates confidence matching and its relationship with accuracy gains. This seems plausible given that individuals tend to weight judgments by decision confidence. By comparing high-trait and low-trait confidence compositions, we aimed to clarify when and why “two heads are better than one.”

## The confidence theory

Bahrami’s et al. (2010) highlighted the critical role of subjective confidence in dyadic decision-making using a perceptual discrimination task with individual and joint responses. Joint responses were more accurate than individual because dyad members shared and used decision confidence as a cue for decision accuracy. We refer to this process as the confidence theory. Subsequent studies by Koriat (2012a, 2015), provided robust support for the confidence theory, demonstrating that dyad members continue to rely on confidence judgments even when they provide a misleading signal, producing a “two heads are worse than one” effect. The confidence theory assumes that people can reliably interpret each other’s confidence, so the response expressed with higher confidence is more likely to be correct. An important extension of this theory is whether dyad members develop a shared confidence scale and align their levels of confidence when working together.

## Confidence matching

Fusaroli et al. (2012) showed that the language used to communicate confidence within dyads converged over time, and that greater alignment predicted larger accuracy gains. Bang et al. (2017) extended this work by proposing confidence matching as a heuristic strategy that dyads use to negotiate influence. In their social condition, participants first responded individually, then their response and confidence rating were displayed on their partner’s computer screen. A joint decision was then automatically selected by taking the higher confidence response. Across trials, individual confidence ratings were more similar in the social condition compared to an isolated condition, and the effect was stronger when feedback was provided. Matching maximized decision accuracy when dyad members had similar ability, but produced smaller gains when ability differed. Because this approach explicitly incentivised confidence matching using an automated decision rule, subsequent work has asked whether matching also emerges under more natural conditions.

Using a multi-phase perceptual task without verbal interaction or joint decisions, Pescetelli and Yueng (2022) observed rapid confidence matching almost immediately, along with a small improvement in decision accuracy. Phases one and three were completed individually and without interaction, while in phase two participants saw their partner’s initial response and confidence rating on their computer screen and observed them update their responses in real-time prior to submitting a final individual response. The authors suggested that confidence matching facilitates more accurate sharing of task-relevant variance in decision confidence while reducing the influence of task-irrelevant variance, such as trait-confidence. They argued that trait confidence is influenced by domain-general factors such as socio-economic background, profession, and personality and that these influences obscure performance-based confidence signals. Although they treated trait confidence largely as noise, it remains an open question whether trait confidence may reflect informative individual differences that dyad members implicitly rely upon when making joint decisions.

Importantly, prior work has conceptualised confidence matching as an adaptive strategy that can help dyads identify the response most likely to be correct. In Bang et al. (2017), non-verbal interaction led individuals to align how they expressed confidence prior to automated joint decisions, functioning as a global strategy for negotiating influence across trials. Confidence was reported once per item, before any interaction, and the mean confidence difference between dyad members was smaller in the social condition than in an isolated condition. In contrast, the present study focused on a more decision-specific form of confidence alignment. Participants provided confidence judgments both before and after interaction on each item, allowing us to examine whether dyad members’ confidence became more similar within a given decision as a result of interaction. We refer to this process as decision-specific confidence matching, to distinguish it from the global form of confidence alignment examined in prior work (e.g., Bang et al., 2017). We further examined decision-specific confidence matching under more naturalistic settings, where dyads communicated verbally and reached joint decisions through consensus.

## The role of trait confidence

Across diverse cognitive tasks (e.g., general knowledge, syllogistic reasoning, perceptual discrimination), a broad trait confidence factor emerges that is positively related to, yet empirically distinct from, cognitive ability. In other words, individuals higher in cognitive ability also tend to report higher confidence across tasks, but the two constructs are not interchangeable. Cognitive ability reflects accuracy of performance of on cognitive tests, whereas trait confidence reflects domain-general self-monitoring and certainty about one’s knowledge and performance. This robust, trait-like tendency has been replicated in numerous studies (e.g., Ais et al., 2016; Johnson, 1939; Kleitman & Stankov, 2001, 2007; Pallier et al., 2002; Soll, 1996; Stankov, 1999; Stankov et al., 2012a; Stankov et al., 2012b).

Trait confidence also shows smaller but consistent associations with other factors. For instance, males tend to report higher confidence than females (Pallier, 2003), and confidence is positively related to the personality trait Openness to Experience (Stankov, 1999), and in some instances it is also related to Optimism Bias, Narcissim, and Extroversion (Ais et al., 2016; Kleitman et al., 2019). In addition, metacognitive impairments affecting confidence judgments are associated with psychiatric conditions involving psychopathology (David et al., 2012; Rouault et al., 2018) as well as heightened experiences of imposter phenomenon (Want & Kleitman, 2006). While these factors contribute to some individual differences in trait confidence, their influence appears secondary to the broader metacognitive mechanisms that underpin the construct.

Recent evidence suggests that decision confidence indexes the likelihood of response replicability rather than accuracy (Koriat, 2024). Specifically, high-confidence judgments tend to be repeated, even if wrong, implying that high-trait confidence individuals may be more decisive yet less flexibile in revising errors. This has important implications for dyads. To illustrate this practically, consider a real-world scenario where two intelligence analysts must collaboratively assess a set of ambiguous and time-sensitive intelligence reports to determine the likelihood of enemy movement in a given area. If both analysts possess high-trait confidence, they may quickly agree on a decisive interpretation and course of action, displaying firm commitment to their initial judgments, even in the face of new or contradictory information. Conversely, if both have low-trait confidence, they may extensively question and reconsider each other’s assessments, potentially leading to indecision or delayed responses. In a mixed-trait confidence dyad, the higher-trait confidence analyst may dominate the decision-making process, strongly advocating for their interpretation while potentially overlooking critical insights from their lower-confidence partner, who remains more open to alternative viewpoints. Thus, trait confidence may influence both the process and outcome, and may limit the advantages of collaboration even among partners with similar ability.

This inflexibility among high-trait confidence members may partly account for the observed asymmetry in confidence matching, where the lower-confidence member typically increases their decision confidence more than the higher-confidence member decreases theirs (Schneider et al., 2024). This asymmetry may inflate overall dyadic confidence, potentially increasing miscalibration between dyadic decision confidence and accuracy. This could account for the widely observed phenomenon that dyads typically report higher-decision confidence than individuals (Patalano & LeClair, 2011; Savadori et al., 2001; Sniezek & Henry, 1989; Zarnoth & Sniezek, 1997), often independent of a corresponding improvement in decision accuracy (Blanchard et al., 2020; Heath & Gonzalez, 1995; Minson & Mueller, 2012; Schuldt et al., 2017). Verbal communication tends to amplify confidence more than non-verbal communication (Mahmoodi et al., 2013).

Two recent studies directly examined how decision confidence changes as a function of the trait confidence composition of dyads. Controlling for cognitive ability, Schuldt et al. (2017) paired individuals with high-trait and low-trait confidence to form dyads of either high-trait, low-trait, or mixed-trait confidence. They found that dyads consisting of two low-trait confidence members had the largest increases in decision confidence. In contrast, Blanchard et al. (2020) found the opposite: dyads with higher-trait confidence showed the largest increases in decision confidence. Both studies used items from the same general knowledge pool, and both reported that these increases in decision confidence did not correspond with improved decision accuracy. The validity of the confidence theory depends on decision confidence reliably tracking decision accuracy. The conflicting findings might stem from differences in measuring trait confidence. Schuldt and colleagues relied on confidence ratings from a single alternate version of their dyadic task which they developed. Notably these ratings were not correlated with accuracy. By contrast, the trait confidence literature typically employs multiple well-validated cognitive tests to reduce measurement error and establish generality (e.g., Kleitman & Stankov, 2007; Stankov et al., 2015; Stankov & Crawford, 1997). In the absence of evidence for convergent and discriminant validity, this operationalisation using a single, self-developed task offers limited support for a domain-general trait. Consistent with the broader literature, Blanchard et al. employed multiple, well-validated cognitive tests, providing a more robust measurement of individual differences in trait confidence.

Despite its evident relevance to dyadic processes and outcomes, the influence of trait confidence on dyadic decision-making remains understudied. Our research directly addressed this gap by investigating how trait confidence moderated changes in decision accuracy, changes in decision confidence, and confidence matching.

## The present study

The present study aimed to replicate and extend previous findings on confidence matching and changes in dyadic confidence. We conducted a pre-screening study to measure trait confidence and cognitive ability, subsequently inviting individuals with high-trait or low-trait confidence to participate in the main study. Participants were paired into high-trait, low-trait, or mixed-trait confidence dyads.

To ensure methodological consistency with previous research on the confidence theory (Bahrami et al., 2010) and confidence matching (Bang et al., 2017; Pescetelli & Yeung, 2022) and to explore its interaction with trait confidence, we included a communication manipulation by varying the type of communication within dyads. In the isolated condition, participants responded to all items twice individually, without any interaction. In the passive condition (similar to Pescetelli & Yeung, 2022), participants initially responded individually and then saw their partner’s answers on their computer screen before making a second individual response. In the active condition, participants first responded individually, then verbally discussed their answers with their partner before jointly deciding on a response.

Verbal interactions allow dyad members to actively question their partner’s reasoning and clarify uncertainties, thereby facilitating a deeper understanding of the underlying rationale behind a response. This richer exchange may enable dyads to better discriminate between responses that are held with higher confidence and are erroneous and those that are held with lower confidence but are accurate. By comparison, the passive viewing condition limited interaction, forcing members to rely exclusively on interpreting numeric confidence ratings without clarification, potentially restricting the precision and accuracy of judgments.

First, we examined whether trait confidence moderated the size of decision accuracy improvements when dyad members transition from individual to dyadic responses while statistically controlling for cognitive ability. Prior studies using a similar design (Blanchard et al., 2020; Schuldt et al., 2017) did not find decision accuracy gains in dyads, possibly because confidence was not a reliable signal for accuracy in their general knowledge tests. In our study, assuming a positive correlation between decision confidence and accuracy, we predicted complex interactions between trait confidence, type of communication, and decision accuracy gains.

For mixed-trait confidence dyads, which were characterised by substantial differences in members’ trait confidence, we expected decision accuracy gains in both the passive and active communication conditions. According to confidence theory, effective collaboration requires accurate assessment and use of each other’s subjective confidence (Bahrami et al., 2010; Bang et al., 2017). However, without explicit feedback like in our study, large initial differences in trait confidence might limit decision accuracy gains more in the passive condition where the transmission of social information is limited and the high-trait confidence member is likely to be more influential (Kerr & Tindale, 2004). The active condition, with verbal discussion and a consensus requirement, should facilitate greater decision accuracy improvements.

### H1a:

 Mixed-trait confidence dyads will show decision accuracy improvements in both passive and active communication conditions, when cognitive ability is statistically controlled.

### H1b:

 Mixed-trait confidence dyads will have greater decision accuracy improvements in the active compared to the passive communication condition, when cognitive ability is statistically controlled.

For dyads with matched levels of trait confidence (both low or both high), we expected accuracy improvements in both passive and active communication conditions because their initially aligned confidence scales should facilitate effective discrimination between response options. Differences were expected based on the consistency of responses and the flexibility of error correction (Koriat, 2024). High-trait confidence individuals may show strong decisiveness and resistance to revising their initial judgments, potentially suppressing the effectiveness of collaboration in the passive condition where they were not required to make joint decisions. Conversely, low-trait confidence dyads may approach interactions with greater openness to new information and more frequent reassessment of initial responses. Thus, low-trait confidence dyads should consistently benefit across both communication conditions.

### H1c:

 Low-trait confidence dyads will improve decision accuracy equally in passive and active communication conditions, when cognitive ability is statistically controlled.

High-trait confidence dyads, characterised by greater decisiveness but reduced flexibility in changing responses when presented with social information, should show limited accuracy gains in the passive condition, where the revision of responses is voluntary. By contrast, the active condition’s requirement for consensus would encourage interrogation of initial judgments, increasing flexibility and the correction of errors, and producing larger decision accuracy gains.

### H1d:

 High-trait confidence dyads will show decision accuracy improvements in both passive and active communication conditions, when cognitive ability is statistically controlled.

### H1e:

 High-trait confidence dyads will have greater decision accuracy improvements in the active compared to the passive communication condition, when cognitive ability is statistically controlled.

Second, we expected to replicate Blanchard et al.’s (2020) finding that dyads higher in trait confidence showed larger increases in decision confidence than those lower in trait confidence. Confidence ratings are bounded, thus, observed gains depend on baseline levels of confidence on a task. For easy items, confidence ratings are often already high, producing a ceiling effect. To mitigate this, we developed a test with moderate difficulty. Following Mahmoodi et al. (2013), we expected greater decision confidence increases in the active communication condition, independent of decision accuracy gains as the mere presence of social information appears to boost decision confidence (e.g., Heath & Gonzalez, 1995).

### H2a:

 Increases in decision confidence will be greater in the active compared to the passive communication condition.

### H2b:

 High-trait confidence dyads will show greater increases in decision confidence compared to low-trait and mixed-trait confidence dyads in both passive and active communication conditions.

Third, we tested whether decision-specific confidence matching would emerge during verbal communication (i.e., active condition). Bang et al. (2017) and Pescetelli and Yeung (2022) demonstrated confidence matching using explicit numeric confidence ratings under communication conditions similar to our passive condition. Fusaroli et al. (2012) showed that there was a large amount of heterogeneity in expressions of confidence for verbally communicating dyads. Dyad members rarely communicated their confidence using numerical values, instead using phrases such as “I’m absolutely sure” or “that was a wild guess.” Their findings showed that expressions of confidence became more similar over time within dyads and greater linguistic alignment of expressions of confidence was associated with greater gains in decision accuracy. These findings provide indirect evidence that decision-specific confidence matching may occur naturally in verbally communicating dyads.

We expected decision-specific confidence matching to be more pronounced in the passive condition. Unlike verbal interactions, which rely on subjective interpretation of diverse verbal expressions of confidence, the passive condition presented confidence as explicit numeric values, making it easier for dyad members to quickly align their confidence judgments and reduce ambiguity about each other’s subjective confidence levels.

### H3a:

 Decision-specific confidence matching will occur in both passive and active communication conditions.

### H3b:

 Decision-specific confidence matching will be stronger in the passive compared to the active communication condition.

Finally, we tested whether decision-specific confidence matching related to improvements in dyadic decision accuracy. Based on Pescetelli and Yeung (2022) and Fusaroli et al. (2012), we expected a positive relationship between decision-specific confidence matching and a dyad’s accuracy gain for both passive and active communication conditions, but we expected a stronger relationship in the passive condition where social information was limited to numerical confidence ratings.

### H4a:

 Decision-specific confidence matching will positively predict a dyad’s decision accuracy improvement in both passive and active communication conditions.

### H4b:

 The relationship between decision-specific confidence matching and a dyad’s decision accuracy gain will be stronger in the passive compared to the active communication condition.

## Method

To examine the role of trait-confidence and type of communication on dyadic outcomes, we first developed three matched versions of a general knowledge test to be administered under the communication conditions. Then we ran a pre-screening study on a large sample of individuals (*N* = 1189) to identify those with high-trait or low-trait confidence to invite for inclusion in the main study (*N* = 210). Our selection criteria aimed to recruit individuals who scored beyond ± 0.50 standard deviations on trait confidence and within ± 1.50 standard deviations of the mean on cognitive ability. To keep the focus on the final stage of this research, descriptions of the general-knowledge test development and the pre-screening study are located in the supplementary materials (Appendices A and B). All stages of the study were approved by the University of Sydney Human Research Ethics Committee (Project Number 2019/706).

## Main study

The main study paired participants together based on their level of trait confidence measured in the pre-screening study and had them complete 3 matched versions of a general-knowledge test each under different communication conditions.

## Participants

Participants were 210 Australian psychology undergraduates (52 males; Mean age = 22.02, SD = 6.12) who completed the study as 105 dyads. All participants previously completed the pre-screening study and half were identified high-trait confidence and the other half as low-trait confidence. Participants were paired to form 35 dyads within each of the trait confidence conditions: low, mixed, and high. They received either partial course credit or financial reimbursement for completing the study.

## Measures

*General Knowledge tests.* The items for the general-knowledge test were sourced from several previous studies (Blanchard et al., 2020; Brewer & Sampaio, 2012; Schuldt et al., 2017; Stankov, 1997). Each version was originally composed of 22 two-alternative forced-choice items; however, due to poor reliability for decision accuracy, they were reduced post-hoc to 10 items. The remaining items covered a broad range of content areas: geography, art, music, film, history, science, and vocabulary. For example, What does the word orthodox mean? Religious or Conventional* (* indicates the correct answer). The resulting internal consistency estimates for each version were around .60. After each item, participants were asked to provide a confidence rating ranging from 50% (guessing) to 100% (completely certain) for the correctness of their response. The internal consistency estimates for confidence were around .80. The three versions were matched on decision accuracy, decision confidence, and content domain. Version one was used in the isolated condition, version two in the passive condition, and version three in the active condition. Because versions were not counterbalanced across communication conditions, we controlled for item differences by using each condition’s individual responses as its baseline. Refer to Appendices A and C of the supplementary materials for more information about the general knowledge tests.

*Esoteric Analogies Test* (EAT; Stankov, 1997): This test was administered in the pre-screening study. It involved participants completing 20 analogies. For each analogy, participants were presented with a pair of words and asked to select one of four options that reflected the same relationship with a target word. For example, LOVE is to HATE as FRIEND is to: (1) LOVER, (2) PAL, (3) OBEY, (4) ENEMY*. Accuracy requires both reasoning skills and prior knowledge thus it is a mixed measure of Fluid Reasoning and Crystallised Intelligence. Prior research with Australian undergraduate samples reported acceptable internal consistency for decision accuracy (ranging from .69 to .74) and excellent internal consistency for confidence (ranging from .88 to .94; Jackson et al., 2016; Law et al., 2022).

*Raven’s Advanced Progressive Matrices* (RAPM; Raven, 1938–65): This test was administered in the pre-screening study. It consists of 36 items, each featuring a 3 × 3 grid of abstract figures forming a horizontal and vertical pattern, with the bottom right figure missing. Participants select one of eight possible options to complete the matrix. Accuracy is a measure of Fluid Reasoning. The internal consistency is excellent for decision accuracy, ranging from .80 to .81, and confidence, ranging from .90 to .92 (Blanchard et al., 2020, 2023). After responding to each item, participants rated their confidence in their answer on a scale from 12.5% (guessing) to 100% (completely certain). In the present study, participants completed a short 15-item version.

*Mini-IPIP* (Donnellan et al., 2006): This questionnaire was administered in the pre-screening study. Participants were presented with 20 statements about their personality, which they rated on a five-point scale ranging from “very inaccurate” (1) to “very accurate” (5). For example, one item asked participants to rate the accuracy of the statement “Am the life of the party.” This scale assesses the Big Five personality traits and has been found to have acceptable internal consistency for Agreeableness (.70), Conscientiousness (.69), Extraversion (.77), Intellect (.65), and Neuroticism (.68).

Several additional individual differences measures were administered to participants as potential covariates, including behavioural inhibition, empathy, motivational traits, psychological safety, risk aversion, social sensitivity, and trust within dyads. The trait confidence conditions did not differ significantly on any of these measures; thus, none were included as covariates in the main analyses. Detailed descriptions of these individual differences measures and the between-group comparisons are provided in Appendix D of the supplementary materials.

## Procedure

Each session involved two participants forming a dyad, who completed the two-hour study online via Zoom. Participants were required to keep their cameras and microphones on throughout the session to ensure compliance with instructions. All tasks were completed using participants’ own computers through a web browser. Dyads were paired based on their trait-confidence levels, which were measured in a pre-screening study. The three communication conditions were presented in blocks. Because the passive condition required that partners had no prior information about each other’s performance, the passive condition always preceded the active condition. Full counterbalancing was therefore not possible. Participants were randomly assigned to one of two sequences: isolated-passive-active or passive-active-isolated. This partial counterbalancing was designed to mitigate practice and fatigue while preserving the integrity of the passive manipulation.

**Fig. 1:**
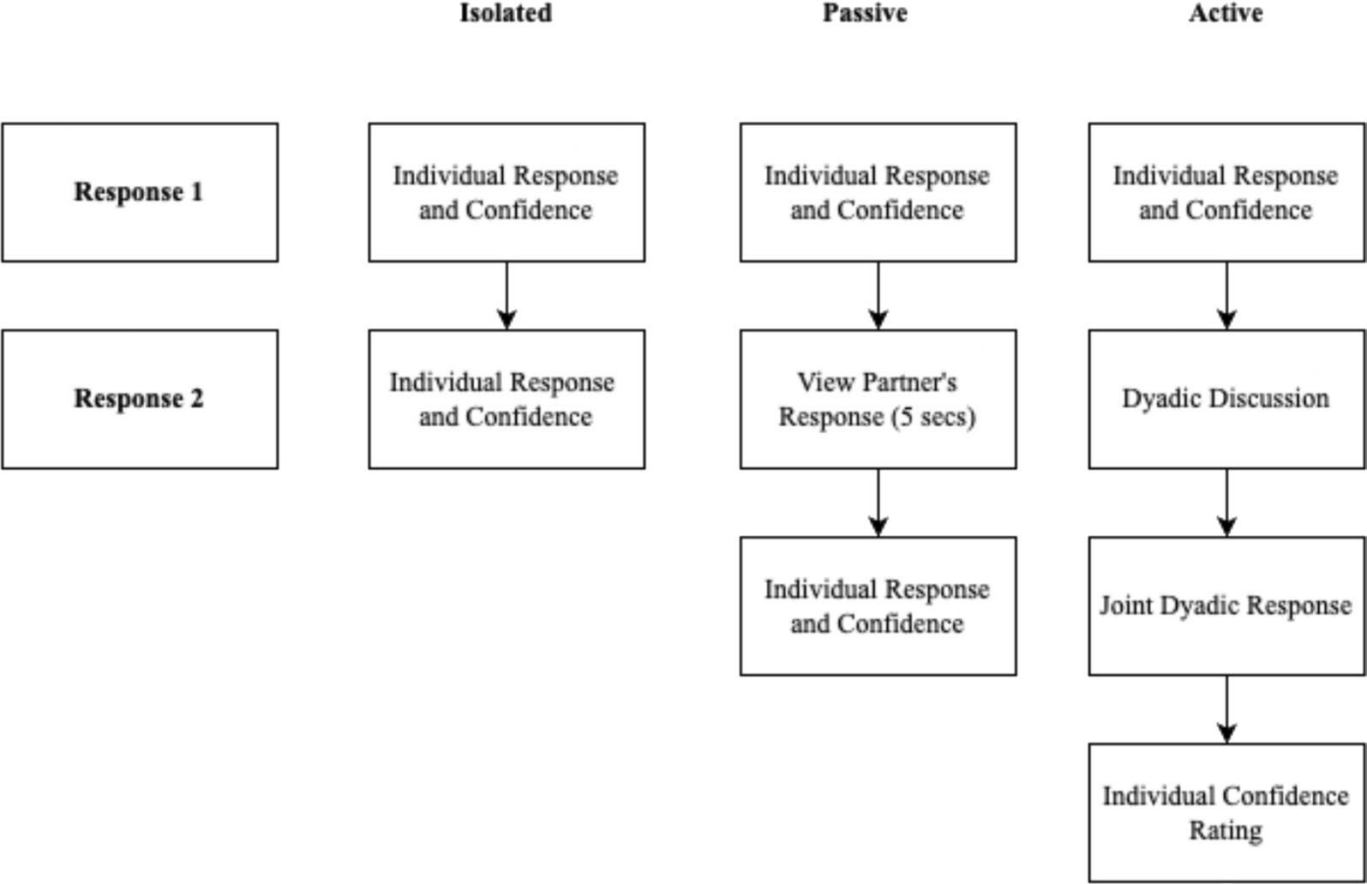
General knowledge test procedure for each communication condition

After providing consent, participants completed the following sequence of measures: demographic questionnaire, Social Motivation Scale, general knowledge tests, Trust Scale, Psychological Safety Scale, Empathy Quotient, Reading the Mind in the Eyes, BIS BAS, and risk aversion. Participants completed each general knowledge test item twice. The first response was always given individually, with no communication permitted between dyad members. The second response, referred to as the “dyad” response, varied by the communication condition. Each trial functioned as follows. First, both participants viewed the same item and independently submitted their responses using a mouse or keyboard. After submitting an initial response, participants individually rated their confidence. For the isolated condition, participants independently repeated this response and confidence rating procedure a second time without any interaction. For the passive condition, after submitting their initial individual response and confidence rating, each participant waited for their partner to finish responding. Once both individual responses were submitted, each participant viewed their partner’s response and confidence rating on screen for 5 s. Following this exposure, participants independently provided a second response to the same question. For the active condition, after both dyad members had submitted their individual responses and confidence ratings, participants pressed the spacebar to view the same item again. They were instructed to discuss the question and agree upon a joint response. Responses were not displayed on their screens like in the passive condition. After discussing, participants agreed on a joint response, both then independently submitted the agreed answer and individually rated their confidence in it. There was no time limit, and dyad members had to submit a joint response before proceeding to the next question, where the process repeated. This process is displayed in Fig. 1.

## Statistical analyses

Unless otherwise specified, we used linear mixed-effects models (LMMs) to account for the hierarchical structure and non-independence of the data. Non-independence is inherent to repeated-measures designs, particularly when individual and dyadic responses are collected from the same participants. In our data, individuals were nested within dyads which were nested within communication conditions. All analyses for decision accuracy and decision confidence were conducted at the individual level to preserve within dyad variance. Due to the method of operationalising decision-specific confidence matching, all analyses were conducted at the dyad level. In each analysis, we systematically compared alternative random effects structures and selected the best-fitting model based on model fit indices. Detailed model specifications and comparisons are reported in the supplementary materials (Appendices E, G, and I), while only the final models are reported in the main text. All analyses were conducted using R version 4.4.2. The LMMs were estimated using the lme4 and lmerTest packages (Bates et al., 2015; Kuznetsova et al., 2017). Because overall model estimates did not provide the specific comparisons required to test our hypotheses, we conducted pairwise contrasts. All *p*-values were adjusted using the Holm procedure to control the type I error rate.

## Results

The results of the main study are reported here, with the results of the pre-screening study reported in the supplementary materials (Appendix B). Both individual and dyadic responses were represented by each participant’s average score across items, allowing comparisons across experimental conditions. This approach allowed us to account for within-dyad variance and the nested structure of the data by modelling random effects for individuals nested within dyads and communication conditions.

## Descriptive statistics

*Decision accuracy and Decision confidence.* Table 1 presents the descriptive statistics, and internal consistency estimates for individuals and dyads for each type of communication and trait confidence condition. Internal consistency was measured using Omega total because we assumed unidimensionality but not tau-equivalence (McDonald, 1999).

**Table 1:** Descriptive statistics and internal consistency estimates for decision accuracy and decision confidence for grouping, communication, and trait confidence conditions (*N* = 210; *n* = 70 per trait confidence condition)

	Individuals				Dyads			
		Low	Mixed	High		Low	Mixed	High
Outcome	ω_t_	Mean (SD)	Mean (SD)	Mean (SD)	ω_t_	Mean (SD)	Mean (SD)	Mean (SD)
*Decision Accuracy*								
Isolated	.56	72.57 (18.31)	77.57 (14.59)	75.29 (19.39)	.55	74.00 (17.89)	77.14 (15.52)	74.86 (18.08)
Passive	.64	74.26 (15.86)	76.71 (16.48)	75.14 (19.62)	.55	82.94 (11.73)	81.29 (14.64)	81.00 (16.08)
Active	.56	72.43 (17.48)	73.86 (17.55)	75.14 (17.51)	.53	79.00 (13.64)	84.14 (12.80)	85.57 (10.85)
*Decision confidence*								
Isolated	.82	67.72 (10.00)	71.60 (10.41)	71.97 (10.27)	.83	66.80 (10.14)	70.44 (10.59)	71.43 (10.27)
Passive	.79	68.22 (8.43)	71.38 (9.96)	71.25 (11.24)	.80	71.53 (8.03)	75.03 (9.73)	75.46 (11.10)
Active	.81	64.15 (9.24)	66.08 (9.32)	67.90 (10.21)	.84	65.79 (8.99)	68.10 (9.14)	71.88 (11.11)

ω_t_ = internal consistency measured using omega total

Internal consistency estimates for decision accuracy were low (Omega total ranged from .53 to .64). While these values raise concerns about measurement precision, they were considered minimally acceptable given the exploratory nature of the research. Nonetheless, caution is warranted when interpreting the findings for decision accuracy. For decision confidence, internal consistency estimates were good (.79 to .84).

We examined the relationship between decision confidence and accuracy for individual responses to assess whether decision confidence was a reliable signal for correctness. Across all three communication conditions, the average within-individual correlation was positive, indicating that confidence generally tracked accuracy. However, the strength of this relationship differed by condition. The passive condition had a significantly stronger correlation (*r* = .44, *p* < .001, 95% CI = [.40-.48]) than both the isolated (*r* = .32, *p* < .001, 95% CI = [.28-.36]) and active (*r* = .27, *p* < .001, 95% CI = [.23-.31]) conditions, which did not significantly differ from each other. These results suggest that decision confidence was a more informative signal for decision accuracy in the passive communication condition and this difference may have influenced our findings. Finally, we checked that the relationship between individual decision confidence (measured in the main study) and trait confidence (measured in the pre-screening study) was as expected. We observed a significant positive correlation between the two confidence measures (*r* = .25, t_208_ = 3.67, *p* < .001), indicating that confidence in the main study was tracking trait confidence.

*Demographic and Individual Difference Measures.* Table 2 displays the descriptive statistics and, where relevant, internal consistency estimates for the demographic, individual difference, and communication variables. Trait confidence and cognitive ability were assessed in the pre-screening study. Trait confidence was computed as each participant’s mean confidence rating across two cognitive ability tests (RAPM and EAT), and cognitive ability was indexed by mean accuracy on the same tests. These prescreen measures were separate from the decision confidence and decision accuracy measures collected in the main study.

**Table 2:** Descriptive statistics and internal consistency estimates for demographic, individual difference, and communication variables for the three trait confidence dyad types (*N* = 210; *n* = 70 per trait confidence condition)

		Low	Mixed	High	
Variable	ω_t_	Mean (SD)	Mean (SD)	Mean (SD)	*F*_*2, 207*_
Age	–	20.79 (6.40)	22.61 (6.73)	22.04 (5.33)	1.60
Proportion of females	–	0.19 (0.39)	0.30 (0.46)	0.26 (0.44)	1.25
Cognitive Ability	–	56.56 (11.12)	62.64 (13.97)	73.38 (10.78)	35.01^***^
RAPM accuracy	.72	50.48 (18.04)	59.71 (19.64)	75.33 (13.82)	36.74^***^
EAT accuracy	.64	62.64 (13.10)	65.57 (14.83)	71.43 (13.62)	7.28^***^
Trait Confidence	–	53.85 (7.40)	68.53 (16.62)	83.30 (5.22)	127.20^***^
RAPM confidence	.91	47.35 (10.57)	64.57 (19.42)	83.02 (8.17)	120.32^***^
EAT confidence	.91	60.35 (10.26)	72.49 (16.48)	83.58 (7.51)	65.47^***^
Agreeableness	.74	3.85 (0.76)	3.91 (0.69)	3.89 (0.72)	0.12
Conscientiousness	.73	3.22(0.88)	3.38(0.84)	3.40(0.86)	0.89
Extraversion	.85	3.01(0.96)	2.90(0.97)	2.65(0.98)	2.49^†^
Intellect	.73	3.53(0.80)	3.79(0.71)	3.88(0.69)	4.24^*^
Neuroticism	.71	3.15(0.77)	3.12(0.86)	3.03(0.90)	0.38

ω_t_ = internal consistency measured using omega total

^***^p < .001, ^*^p < .05, ^†^*p* < .10

The means and standard deviations are as expected given the results of other studies that have used the same measures on undergraduate populations (e.g., Blanchard et al., 2020, 2025; Jackson et al., 2017; Law et al., 2018; Law et al., 2022). Internal consistency estimates ranged from acceptable (.64) to excellent (.91) for all psychological measures. ANOVA tests confirmed that cognitive ability and trait confidence significantly differed between the three dyad types differing on trait confidence conditions. Tukey’s HSD tests indicated that trait confidence and cognitive ability were significantly higher for mixed-trait vs low-trait (*p* < .001 and *p* < .01, respectively), high-trait vs low-trait (*p* < .001 for both), and high-trait vs mixed-trait (*p* < .001 for both) confidence dyads. These differences in trait confidence confirmed our manipulation check, as they were intended by design and resulted from the pre-screening study which identified high-trait (> + 0.50 SD) and low-trait (< -0.50 SD) confidence individuals. Pre-screening also aimed to control for differences in cognitive ability across the trait confidence conditions, however, residual differences in ability remained. To mitigate this, we statistically controlled for cognitive ability by including EAT accuracy and RAPM accuracy as covariates in all analyses for decision accuracy, decision confidence, and decision-specific confidence matching. Lastly, intellect was significantly higher for those in high-trait compared to the low-trait confidence dyads (*p* = .02), but the other conditions did not differ. There were no significant differences between the trait confidence conditions on the other measures.

## Effects of trait confidence and communication on decision outcomes

**Fig. 2:**
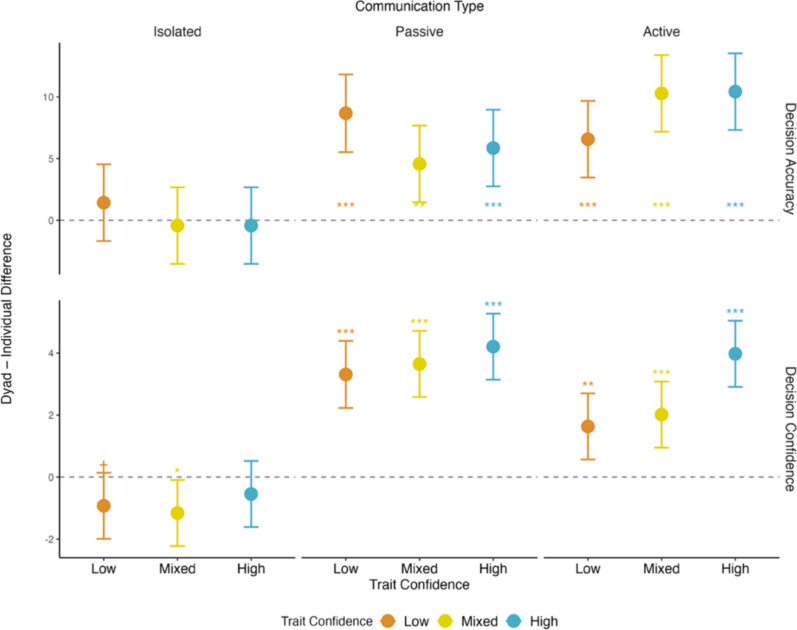
The differences between dyads and individuals on decision accuracy and decision confidence for the communication and trait confidence conditions

We fit a series of LMMs to examine the effects of grouping (individual vs. dyad; within-subjects), type of communication (isolated vs. passive vs. active; within-subjects), and trait confidence (low-trait vs. mixed-trait vs. high-trait; between-subjects) on decision accuracy and decision confidence. We included two cognitive ability estimates (i.e., EAT accuracy and RAPM accuracy) as covariates to control for the effect of ability. The models included random intercepts and slopes for communication across individuals and dyads. Our interpretations focused on the three-way interaction effects. Table 3 presents the fixed effects and Fig. 2 displays the differences between dyads and individuals in each condition for both outcomes.

**Table 3:** Pairwise comparisons for decision accuracy and decision confidence (N = 1256)

			Decision			
			Accuracy		Confidence	
			b	SE	*b*	SE
*Main Effects*						
Grouping	Dyad vs Ind		5.22^***^	0.53	1.80^***^	0.18
Communication	Passive vs Isolated		3.33^*^	1.36	2.24^**^	0.64
	Active vs Isolated		3.12^*^	1.29	− 2.67^***^	0.57
	Passive vs Active		− 0.21	1.33	− 4.91^***^	0.61
Trait Confidence	Mixed vs Low		2.54	2.16	3.14	1.68
	High vs Low		1.67	2.32	4.47^*^	1.81
	High vs Mixed		− 0.87	2.21	1.33	1.72
RAPM Acc			− 0.07^†^	0.04	− 0.06^*^	0.03
EAT Acc			0.22^***^	0.04	0.15^***^	0.03
*Three-Way Simple Effects Interactions*						
Trait Confidence	Communication	Grouping				
Low	Isolated	Dyad vs Ind	1.43	1.58	− 0.92^†^	0.54
Mixed	Isolated	Dyad vs Ind	− 0.43	1.58	− 1.16^*^	0.54
High	Isolated	Dyad vs Ind	− 0.43	1.58	− 0.54	0.54
Low	Passive	Dyad vs Ind	8.68^***^	1.6	3.31^***^	0.55
Mixed	Passive	Dyad vs Ind	4.57^**^	1.58	3.65^***^	0.54
High	Passive	Dyad vs Ind	5.86^***^	1.58	4.21^***^	0.54
Low	Active	Dyad vs Ind	6.57^***^	1.58	1.63^**^	0.54
Mixed	Active	Dyad vs Ind	10.29^***^	1.58	2.02^***^	0.54
High	Active	Dyad vs Ind	10.43^***^	1.58	3.98^***^	0.54

^***^p < .001, ^**^p < .01, ^*^p < .05, ^†^*p* < .10

*N* = 210 participants × 2 grouping conditions × 3 communication conditions—4 missing data points = 1256

## Baseline analyses

First, we examined baseline differences between the conditions by comparing individual responses between the communication and trait confidence conditions. This allowed the baseline tests and main tests to be conducted within a single model thus maintaining control over the Type I error rate. The results of all baseline comparisons are presented in Table F2 in Appendix F of the supplementary materials.

*Decision accuracy.* No significant differences were found between the conditions on decision accuracy at baseline (individual responses).

*Decision confidence.* Baseline analyses showed significant differences on decision confidence between the communication conditions. Specifically, individuals in the active communication condition had lower decision confidence than those in the passive and isolated communication conditions across all three trait confidence conditions. Given that these differences existed prior to, and cannot be attributed to, the grouping intervention, we did not perform between-group simple effects contrasts comparing the communication conditions at each level of grouping or trait confidence.

## Main analyses

### Decision accuracy

The overall model for decision accuracy significantly differed from the null model (χ^2^ = 176.28, *p* < .001, R^2^ = .09). Together, the fixed effects accounted for 9% of the variance in decision accuracy.

*Three-Way Interactions.* For mixed-trait confidence dyads and in support of hypothesis 1a, collective judgments were significantly more accurate than individual judgments in the passive condition (*b* = 4.57, SE = 1.58, *t*_619_ = 2.89, *p* < .01) and active communication conditions (*b* = 10.29, SE = 1.58, *t*_619_ = 6.51, *p* < .001). In support of hypothesis 1b, the magnitude of decision accuracy gains for mixed-trait confidence dyads was significantly greater in the active compared to the passive communication condition (*b* = 5.71, SE = 2.24, *t*_619_ = *2.56*, *p* = .01). In support of hypothesis 1c, low-trait confidence dyads were significantly more accurate than individuals in the passive condition (*b* = 8.68, SE = 1.60, *t*_*619*_ = 5.41, *p* < .001) and the active condition (*b* = 6.57, SE = 1.58, *t*_*619*_ = *5.41*, *p* < .001), and the size of the decision accuracy improvements did not differ between the active and passive conditions (*b* = -2.11, SE = 2.25, *t*_619_ = *-*1.58, *p* = .11). In support of hypothesis 1d, high-trait confidence dyads were significantly more accurate in their collective judgments than individually for both the passive (*b* = 5.86, SE = 1.58, *t*_619_ = 3.70, *p* < .001) and active communication conditions (*b* = 10.43, SE = 1.58, *t*_619_ = 6.60, *p* < .001). In support of hypothesis 1e, the magnitude of the decision accuracy improvement was significantly larger in the active than the passive communication condition (*b* = 4.57, SE = 2.24, *t*_619_ = 2.04, *p* = .04).

### Decision Confidence

The overall model predicting decision confidence significantly differed from the null model (χ^2^ = 277.60, *p* < .001, R^2^ = .13), with fixed effects explaining 13% of the variance.

*Three-Way Interactions*. For all trait confidence conditions, dyadic judgments showed significantly higher decision confidence than individual judgments in the passive (Low: *b* = 3.31, SE = 0.55, *t*_*619*_ = 6.01, *p* < .001; Mixed: *b* = 3.65, SE = 0.54, *t*_*619*_ = 6.73, *p* < .001; High: *b* = 4.21, SE = 0.54, *t*_*619*_ = 7.76, *p* < .001) and the active communication conditions (Low: *b* = 1.63, SE = 0.54, *t*_*619*_ = 3.01, *p* < .01; Mixed: *b* = 2.02, SE = 0.54, *t*_*619*_ = 3.72, *p* < .001; High: *b* = 3.98, SE = 0.54, *t*_*619*_ = 7.33, *p* < .001). Furthermore, decision confidence gains were significantly larger in the passive than active communication condition for low-trait (Low: *b* = -1.68, SE = 0.77, *t*_*619*_ = -2.17, *p* = .03) and mixed-trait confidence dyads (*b* = -1.63, SE = 0.77, *t*_*619*_ = -2.13, *p* = .03), but there was no difference for high-trait confidence dyads (*b* = -0.23, SE = 0.77, *t*_*619*_ = -0.30, *p* = .76). Thus, contrary to hypothesis 2a, the passive condition was associated with greater increases in decision confidence than the active communication condition for low-trait and mixed-trait confidence dyads, with no difference was observed for high-trait confidence dyads.

Next, to test hypothesis 2b, we compared confidence gains (dyadic – individual) across trait confidence conditions, separately within each communication condition. For the active condition, the increase in dyadic decision confidence was significantly greater for high-trait compared to low-trait (*b* = 2.34, SE = 0.77, *t*_*619*_ = 3.05, *p* < .01) and mixed-trait confidence dyads (*b* = 1.96, SE = 0.77, *t*_*619*_ = 2.55, *p* = .01). No difference was observed between low-trait and mixed-trait confidence dyads (*b* = 0.38, SE = 0.77, *t*_*619*_ = 0.50, *p* = .62). For the passive condition, increases in dyadic decision confidence did not differ as a function of trait confidence (High vs low: *b* = 0.90, SE = 0.77, *t*_*619*_ = 1.16, *p* = .25; High vs mixed: *b* = 0.56, SE = 0.77, *t*_*619*_ = 0.73, *p* = .47; Low vs mixed: *b* = 0.34, SE = 0.77, *t*_*619*_ = 0.44, *p* = .66). In partial support of hypothesis 2b, those in the high-trait confidence condition had the largest dyadic decision confidence gains compared to the low-trait and mixed-trait confidence conditions in the active condition, but there were no differences in the passive condition.

### Decision-specific confidence matching

We examined whether dyads engaged in decision-specific confidence matching, defined as increased similarity between dyad members’ confidence judgments after interaction on a given item. Decision-specific confidence matching was operationalised as the difference between individual confidence ratings (before interaction) minus the difference between dyadic confidence ratings (after interaction) for each item. Positive values indicated greater confidence alignment following interaction, values of zero indicated no change, and negative values indicated greater confidence divergence. We explored whether the extent of decision-specific confidence matching varied by communication condition and trait confidence.

**Fig. 3:**
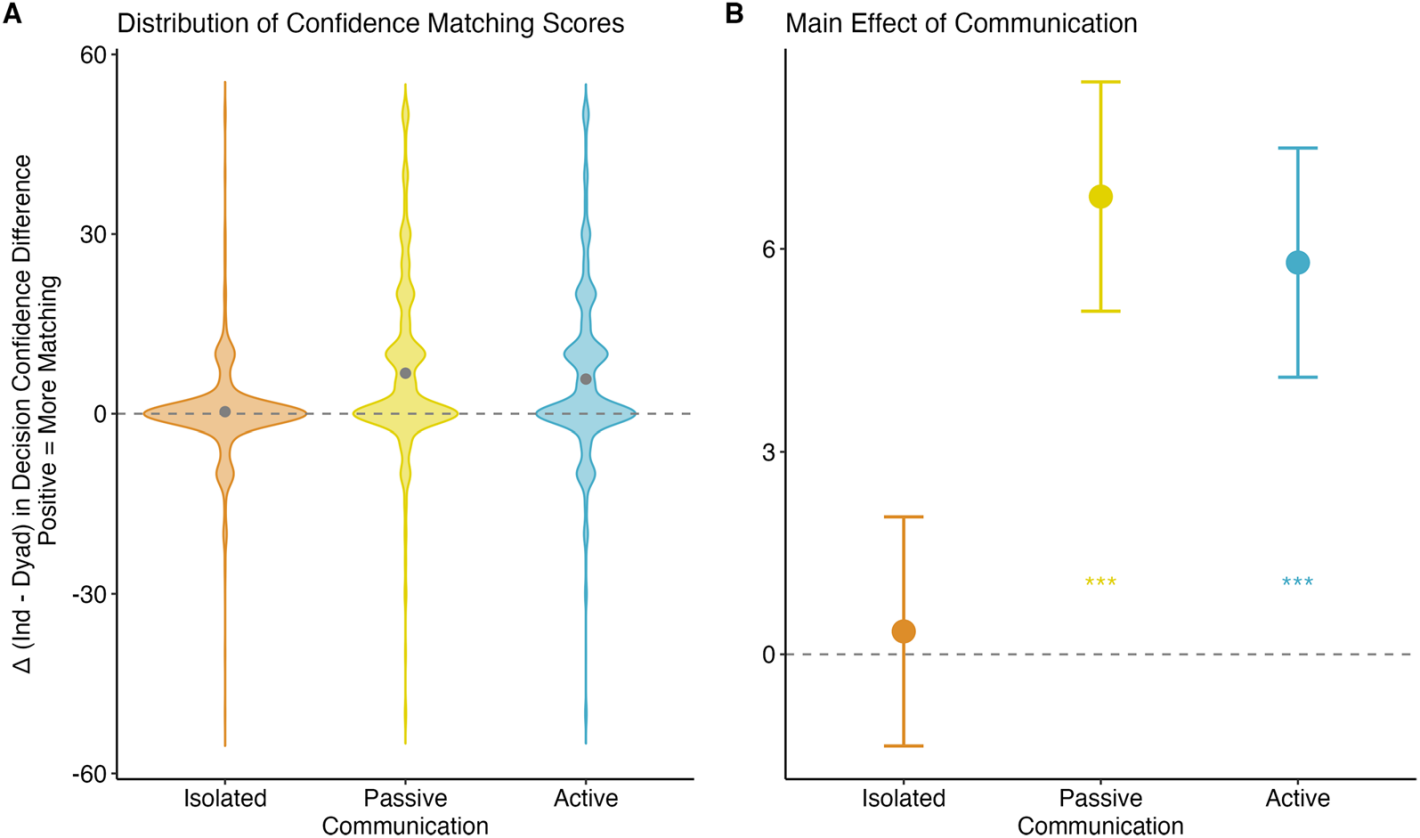
Decision-specific confidence matching **A** distribution plots and **B** mean differences for the communication conditions

We fit a LMM using communication type and trait confidence to predict decision-specific confidence matching while controlling for the two cognitive abilities. None of the interaction effects were significant thus the final model included main effects only and random intercepts for group and item number. More details about the model selection process are available in Appendix G of the supplementary materials. Table 4 presents the fixed main effects and Fig. 3 displays distribution plots and mean levels of decision-specific confidence matching in the communication conditions.

**Table 4:** Main effects for decision-specific confidence matching (*N* = 3140)

*Variable*	Mean	SE	*t*
Isolated	0.34	0.83	0.41
Passive	6.78	0.83	8.16^***^
Active	5.80	0.83	6.98^***^
Low	4.31	0.70	6.12^***^
Mixed	3.90	0.63	6.15^***^
High	4.70	0.73	6.42^***^
RAPM Acc	0.00	0.03	− 0.08
EAT Acc	− 0.01	0.03	− 0.42

^***^p < .001

105 dyads × 3 communication conditions × 10 items—10 missing data points = 3140

The overall model significantly differed from the null model (χ^2^ = 26.84, *p* < .001, R^2^ = .05), accounting for 5% of the variance in decision-specific confidence matching. Results indicate that the level of decision-specific confidence matching in the passive (Mean = 6.78, SE = 0.83, *t*_29_ = 8.16, *p* < .001) and active (Mean = 5.80, SE = 0.83, *t*_29_ = 6.98, *p* < .001) communication conditions was significantly greater than zero. In contrast, decision-specific confidence matching did not occur in the isolated condition (Mean = 0.34, SE = 0.83, *t*_29_ = 0.41, *p* = .68). Contrasts also revealed that the level of decision-specific confidence matching in the passive (Mean difference = -6.44, SE = 1.15, *t*_27_ = -5.59, *p* < .001) and active conditions (Mean difference = -5.46, SE = 1.15, *t*_27_ = -4.74, *p* < .001) was significantly greater than the isolated condition. However, passive and active did not differ from each other (Mean difference = 0.978, SE = 1.15, *t*_27_ = 0.85, *p* = 0.40).

In support of hypothesis 3a, decision-specific confidence matching emerged in both the passive and active conditions. However, the magnitude of decision-specific confidence matching effects did not differ between the passive and active conditions, thus, hypothesis 3b was not supported.

**Fig. 4:**
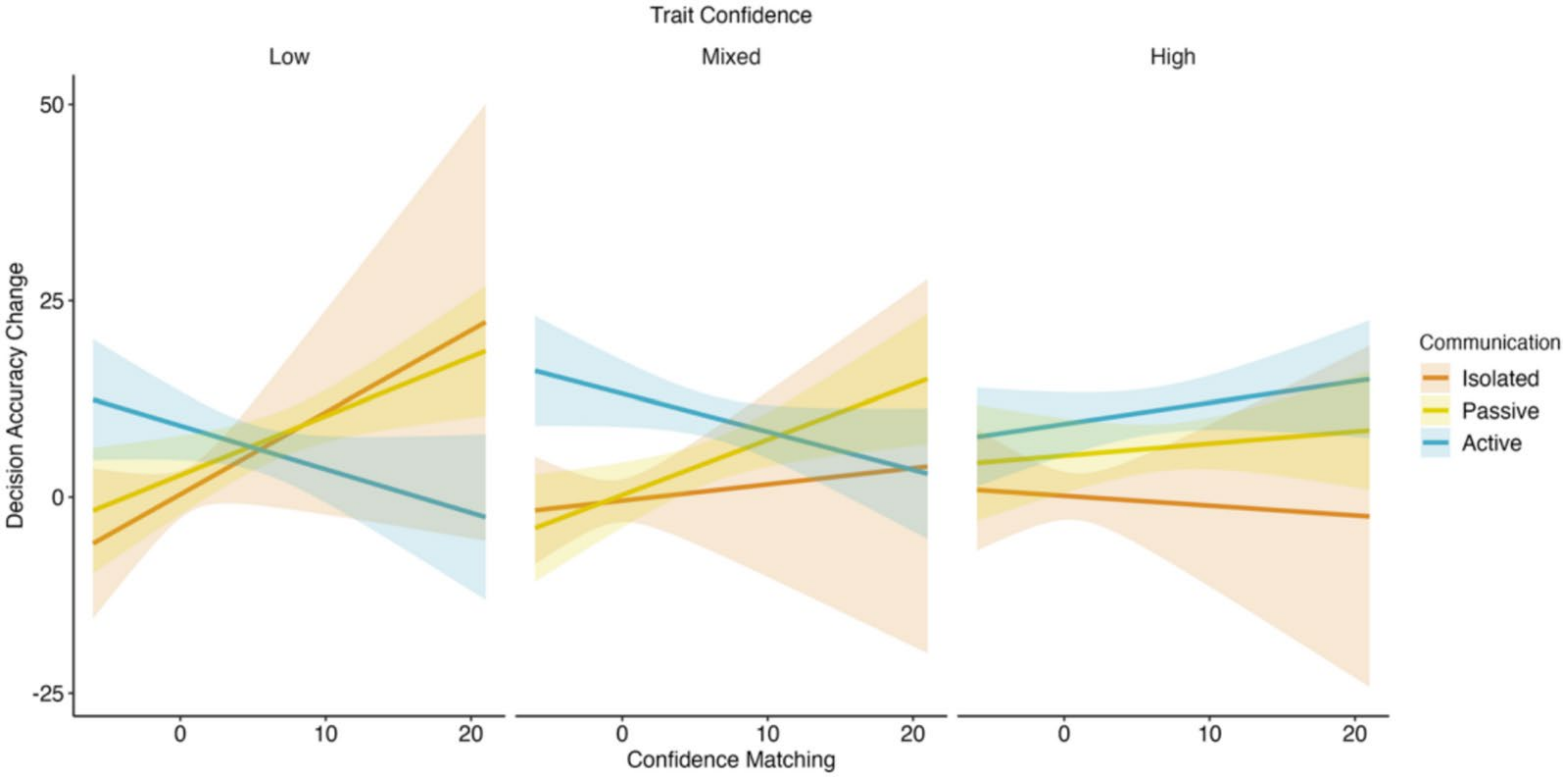
Effect of decision-specific confidence matching on dyadic accuracy change by communication and trait confidence

Next, we investigated whether decision-specific confidence matching predicted the change in a dyad’s decision accuracy at the aggregate level. For each dyad, we computed the mean level of decision-specific confidence matching and the mean change in decision accuracy (difference between overall dyadic accuracy and individual accuracy). We then entered decision-specific confidence matching, communication, trait confidence, and the two cognitive ability covariates into the model to predict the change in decision accuracy. The final model included fixed effects only and the three-way interaction effect. Table 5 presents the fixed effects. Full model details are provided in Appendix J of the supplementary materials. Figure 4 illustrates the modelled slopes for the relationship between decision-specific confidence matching and the change in decision accuracy as a function of communication and trait confidence.

**Table 5:** Interaction effects of decision-specific confidence matching predicting the change in decision accuracy (*N* = 324)

Communication	Condition	Decision Accuracy Change		
		*b*	SE	*t*
Isolated	Low	1.04	0.69	1.51
Isolated	Mixed	0.21	0.56	0.37
Isolated	High	− 0.13	0.54	− 0.23
Passive	Low	0.75	0.28	2.64^**^
Passive	Mixed	0.70	0.27	2.65^**^
Passive	High	0.15	0.26	0.59
Active	Low	− 0.55	0.32	− 1.70^†^
Active	Mixed	− 0.48	0.27	− 1.80^†^
Active	High	0.27	0.23	1.17

^**^p < .01, ^†^*p* < .10

The full model was significant (*F*_19,294_ = 5.58, *p* < .001, R^2^ = .27), accounting for 27% of variance in the decision accuracy change. Removing the confidence-matching term reduced R^2^ to .20, indicating that decision-specific confidence matching uniquely explained 7% of the variance. We focused on the significant three-way interaction (*F*_*4,294*_ = 2.79, *p* = .03). In the passive condition, decision-specific confidence matching was a significant positive predictor of decision accuracy gains for low-trait (*b* = 0.75, SE = 0.28, *t*_294_ = 2.64, *p* < .01) and mixed-trait confidence dyads (*b* = 0.70, SE = 0.27, *t*_294_ = 2.65, *p* < .01), but not high-trait dyads (*b* = 0.15, SE = 0.26, *t*_294_ = 0.60, *p* = .55). No significant relationships were observed in the isolated or active conditions. Furthermore, the matching-accuracy slope was significantly stronger in the passive than active for low-trait confidence (*t*_294_ = 3.02, *p* < .01) and mixed trait confidence dyads (*t*_294_ = 3.14, *p* < .01), but not high trait confidence dyads (*t*_294_ = 0.35, *p* = 1.00). This partially supports hypothesis 4a, however, passive did not significantly differ from isolated for low-trait (*t*_294_ = 0.39, *p* = 0.70) or mixed-trait confidence dyads (*t*_294_ = -0.80, *p* = 0.54). Furthermore, the matching-accuracy slopes did not differ by trait confidence within any communication condition.

## Discussion

In the present study, we examined how trait confidence and the type of communication influenced decision accuracy, decision confidence, and decision-specific confidence matching in dyads. By statistically controlling for cognitive ability, we ensured that observed effects primarily reflected differences in trait confidence. This study offers several novel contributions: 1) we demonstrated that trait confidence moderates dyadic gains in both decision accuracy and confidence; 2) we observed that these moderating effects depend on the mode of communication (passive vs active); and 3) we showed that decision-specific confidence matching naturally emerges when dyads engage in realistic verbal interactions.

## Decision accuracy: interaction of communication and trait confidence

Consistent with prior research, we observed a “two heads are better than one” effect when dyads were allowed to communicate (both passive and active; e.g., Bahrami et al., 2010; 2012a; Koriat, 2015). Importantly, we extended previous findings by showing that the size of this dyadic advantage depended on both the dyad’s trait confidence composition and the type of communication. Specifically, mixed-trait and high-trait confidence dyads demonstrated larger accuracy improvements in the active communication condition, whereas low-trait confidence dyads benefitted equally from passive and active communication. These effects are unlikely to reflect differences in cognitive ability because we statistically controlled for it in our analyses, observed no baseline accuracy differences between trait confidence conditions, and found comparable overall gains across trait confidence conditions, albeit under different communication conditions.

For mixed-trait confidence dyads, our findings are consistent with the idea that large initial discrepancies in decision confidence can limit the potential gains from information pooling, particularly during disagreement. Under passive communication, where members can view each other’s answers but cannot clarify or negotiate, such discrepancies may hinder effective information integration, especially if the high-trait confidence member dominates decisions. In contrast, active verbal communication may reduce this asymmetry, likely by compelling high-trait confidence members to engage more openly with their partner’s perspective. In our analyses, we did not model trial-level trajectories. Instead, we related each dyad’s pre-post interaction change in the confidence gap to its dyad-individual accuracy gains. This interpretation aligns with Bang et al. (2017), who proposed that matching reduces asymmetry and improves selective weighting, and with Koriat’s (2024) view that confidence reflects response replicability, where high-trait confidence individuals may rigidly adhere to initial judgments unless prompted to revise them through richer social interaction and consensus.

Dyads composed of similarly confident members showed distinct patterns. Low-trait confidence dyads achieved consistent accuracy gains across both communication types, suggesting a general openness to external input and greater willingness to revise initial judgments (Koriat, 2024). By contrast, high-trait confidence dyads, showed larger accuracy improvements under active verbal discussion, likely because verbal interaction forced reconsideration of their judgments. Under passive conditions, high-trait dyads may have discounted their partner’s input, relying more heavily on their own judgments.

Together, these results align with the confidence theory (Bahrami et al., 2010); however, because we lacked a condition where participants shared decisions without confidence, we could not isolate the unique contribution of confidence information from the knowledge of a partner’s choice. Our results also suggest that trait confidence shapes how communication influences joint accuracy. Verbal communication appears more important when a dyad contains at least one high-trait confidence member. Considering trait confidence could help improve dyadic decision-making outcomes.

## Decision confidence: passive gains and active uncertainty

We replicated a well-established finding that dyads have higher-decision confidence than individuals (e.g., Sniezek & Henry, 1989; Zarnoth & Sniezek, 1997). Importantly, this increase in confidence was not uniform across dyads. Consistent with our previous finding (Blanchard et al., 2020), we found that trait confidence moderated dyadic confidence gains but only under active communication. Specifically, dyads composed of high-trait confidence members showed the largest increases in decision confidence during verbal discussion. This contrasts with Schuldt et al. (2017), who reported greater confidence gains for low-trait confidence dyads. This discrepancy likely stems from methodological differences in measuring trait confidence. Our approach, like Blanchard et al. (2020), employed multiple validated cognitive tests that reliably captured stable, domain-general individual differences in trait confidence. Alternatively, Schuldt and colleagues used a single matched version of their general knowledge test with no correlation between decision accuracy and confidence. Their measure may not have been tapping into the confidence trait.

Our findings suggest a nuanced relationship between communication, trait confidence, and dyadic confidence gains. Low-trait and mixed-trait confidence dyads experienced larger increases in decision confidence in the passive than the active condition. Whereas high-trait confidence dyads showed similarly sized increases in decision confidence in the passive and active conditions. This pattern suggests that verbal discussion makes uncertainty more salient, as it requires dyad members to consider alternative viewpoints and openly debate (Tindale & Sheffey, 2002). Low-trait confidence members, who are generally less decisive (Jackson et al., 2017), may be especially sensitive to this explicit uncertainty, potentially dampening their decision confidence in the active communication condition.

This interpretation aligns with prior work by Pescetelli and Yeung (2020), who found that decision confidence increases following initial agreement and decreases after disagreement. In our task, and similar to prior studies, dyads agreed on a large majority of trials (Koriat, 2015 reported 82% agreement; our sample was similar), so high levels of agreement likely amplified overall decision confidence. However, active communication may highlight the presence of disagreement more clearly than passive communication, especially for low-trait confidence individuals. We speculate that trait confidence may moderate how dyads respond to agreement and disagreement at the item-level under active communication. Our results suggest that high-trait confidence individuals may increase more in decision confidence when agreement occurs, and low-trait confidence members may decrease in decision confidence more when disagreement occurs.

Two key insights emerge from our findings. First, passive communication may boost dyadic confidence more than active discussion by obscuring awareness of disagreement. Second, trait confidence moderates how dyads respond to collaboration, shaping both the magnitude and direction of confidence gains. These findings refine our understanding of when and why dyadic confidence is amplified and highlights the importance of considering individual differences in trait confidence in models of collaborative decision making.

## Decision-specific confidence matching: communication mode matters

We found evidence that decision-specific confidence matching naturally occurs under both passive and active communication, but its predictive value for accuracy gains was small and context dependent. In the passive condition, decision-specific confidence matching positively predicted the dyad-individual accuracy change for low-trait and mixed-trait confidence dyads. However, these slopes did not differ from the isolated condition, where members never interacted. Thus, although the passive slopes were statistically different from zero, we cannot conclude they were stronger than simple retest effects. Together with prior work (e.g., Pescetelli & Yeung, 2022), this suggests the relationship between decision-specific confidence matching and accuracy gains is small and may be most evident in non-verbal contexts where numeric confidence is made explicit.

Why might decision-specific confidence matching emerge more clearly in the passive than the active condition? First, the nature of communication differed distinctly between the conditions. Passive communication explicitly provided numeric confidence values, offering a precise decision heuristic. Active communication involved richer verbal discussion but more ambiguous communication of decision confidence as verbally interacting dyads rarely communicate confidence numerically (Fusaroli et al., 2012). Thus, despite rapid confidence alignment in both, numeric decision-specific confidence matching may have greater predictive power due to its precision and clarity. Second, the baseline correlation between decision confidence and accuracy was stronger in the passive (*r* = .44) than in the active (*r* = .27) condition. One likely contributor is measurement as accuracy had higher reliability in the passive than the active condition (ω = .64 vs .56), so less measurement error would produce a larger correlation. Order may also have played a role, as the passive block always preceded the active block, making practice or fatigue plausible influences. Finally, task expectations differed. In the passive condition participants knew their individual responses would be shown to their partner, which may have prompted more careful metacognitive appraisal than in the other conditions where individual responses were not displayed. Future work could address order effects with a between-subjects design and test whether stronger baseline confidence-accuracy correlations amplify the effectiveness of decision-specific confidence matching (Hermans et al., 2025). Lastly, our brief task and lack of feedback likely limited the strength of decision-specific confidence matching effects, particularly under more complex verbal interactions. Longer tasks, like those used by Bang et al. (2017) and Pescetelli and Yeung (2022), might yield clearer effects, especially as dyads tend to develop a shared mostly non-numeric language for verbal expressions of confidence (Fusaroli et al., 2012).

Interestingly, although decision-specific confidence matching predicted decision accuracy gains in the passive condition, dyads in the active condition demonstrated larger overall accuracy improvements. This suggests that mechanisms beyond confidence alignment played substantial roles in the active condition. For example, argument quality has been shown to overcome the influence of confidence in collective decisions (Trouche et al., 2014). These are important considerations for future studies.

## Implications and contributions to theory

Our findings have important theoretical implications for the confidence matching literature. For example, Pescetelli and Yeung (2022) raised the “similarity hypothesis” which states that observed confidence matching could reflect pre-existing similarities rather than interaction driving convergence. Our results with mixed-trait confidence dyads provide direct evidence that decision-specific confidence matching naturally emerges due to social interaction rather than pre-existing similarity. Despite these dyads comprising members with substantially different baseline levels of trait confidence, decision-specific confidence matching occurred only in conditions that permitted interaction. Our findings also offer insights into the “two heads are better than one” effect by demonstrating that trait confidence significantly moderates dyadic accuracy benefits, and decision confidence gains through communication. This suggests trait confidence may influence how effectively dyads collaborate, share information, and possibly calibrate their confidence judgments during collective tasks. Lastly, our findings hold important implications for human-AI collaboration. Recent research indicates that large language models display overconfidence when faced with novel or challenging problems not well represented in their training data (Phan et al., 2025). This tendency could limit their effectiveness in collaborative decision-making contexts where decision accuracy is critical. However, research also shows that hybrid collectives combining human and AI inputs typically outperform both human-only and AI-only groups (Zöller et al., 2024). Our study contributes to this emerging literature by suggesting that individual differences in human trait confidence might further optimise such hybrid collaborations. Specifically, pairing humans who have complementary levels of trait confidence with AI systems may mitigate the AI’s overconfidence by introducing beneficial uncertainty, thus promoting more accurate and calibrated joint decisions. Future research should explore whether human trait confidence influences the outcomes of human-AI collaboration.

## Practical implications

Our findings have several practical implications for organizations, managers, and groups. For high-trait and mixed-trait confidence dyads, active verbal communication maximized dyadic accuracy, whereas low-trait confidence dyads performed equally well with simpler passive communication. Given that passive communication requires fewer organizational resources (i.e., shorter meetings, reduced logistical overhead, and lower financial costs) and fewer individual resources (i.e., less time spent on task and lower cognitive load), passive communication may be ideal for low-trait confidence dyads. Conversely, high-trait and mixed-trait confidence dyads face a resource-accuracy trade-off, with active communication enhancing decision accuracy but requiring additional time and effort. Managers could optimize dyadic performance and resource allocation by strategically pairing individuals according to trait confidence and selecting suitable communication methods. Passive communication may be sufficient for high-trait or low-trait confidence dyads when maximizing decision accuracy is less critical, but active verbal discussion is necessary for mixed-trait confidence dyads to reliably achieve the benefits of collective decision making.

## Limitations and future directions

Several limitations highlight areas of focus for future research. First, although our general knowledge tests provided valuable insights, their internal consistency for decision accuracy was low (.53-.64) which suggests that caution is warranted when interpreting the associated findings. The heterogenous range of topics (e.g., art, history, geography, science, film) may have contributed to the lower reliability. Future studies should employ validated measures of general knowledge to enhance reliability and replicate our findings.

Second, our general knowledge tests contained only 10 items each. Although confidence matching emerges rapidly (Pescetelli & Yeung, 2022), the brevity of our tests may have constrained the extent to which it is related to decision accuracy, especially under active communication. The shortness also prevented an analysis of agreement and disagreement trials. Disagreement is where revision is most likely and where confidence often decreases, whereas agreement tends to increase confidence. Future studies should use longer tasks to give dyads more time to calibrate their confidence scales and to permit examination of the effects of agreement.

Third, although our attempt to disentangle the relationship between trait confidence and cognitive ability was unsuccessful in the pre-screening study, the process restricted variability in cognitive ability to approximately ± 1.50 SD of the mean. This limits generalisability because a greater distribution of cognitive ability scores exists for those with high and low trait confidence in the broader population. Dyads with more pronounced ability differences might experience outcomes that differ from our findings, particularly given that substantial ability differences can undermine dyadic benefits (e.g., Bahrami et al., 2010; Bang et al., 2017). Future studies should explore how cognitive ability differences interact with trait confidence in dyadic decision-making.

Fourth, the weaker confidence-accuracy correlation and lower individual confidence in the active condition may have influenced results for decision confidence and decision-specific confidence matching. Several factors may have contributed to this: lower measurement reliability for accuracy in the active compared to passive condition (ω = .56 and 64, respectively), order effects because the passive condition always preceded the active condition, and differing task expectations as individual confidence was displayed to the partner in the passive but not in the active condition. Future studies should employ a between-subjects design so each participant completes one condition and counterbalance items across conditions to remove item-content confounds.

Lastly, our tests involved non-misleading items where the confidence-accuracy correlation is typically positive. An interesting direction for future research would be to examine whether decision-specific confidence matching emerges for misleading items, where confidence negatively relates to decision accuracy. This could help to further delineate the boundary conditions for confidence matching as an effective strategy.

## Conclusion

Our findings extend our understanding of dyadic decision-making by characterising how individual differences in trait confidence and communication jointly shape dyadic decision accuracy, decision confidence, and decision-specific confidence matching. We found evidence that trait confidence moderates both decision accuracy and confidence gains in dyadic decision making, especially under conditions enabling active verbal communication. We also demonstrated that decision-specific confidence matching occurs naturally when verbal interaction is permitted, extending prior research beyond artificial numeric confidence conditions into more realistic verbal communication contexts. Critically, we found that while decision-specific confidence matching predicted decision accuracy improvements for passive communication with just numeric confidence ratings, richer verbal interactions with more ambiguous confidence expressions produced greater decision accuracy gains, likely through other mechanisms. Our findings highlight the relationship between decision-specific confidence matching and decision accuracy as small, nuanced, and likely contingent upon the method of communicating confidence. By demonstrating the importance of trait confidence and communication mode, our research may inform future recommendations for enhancing dyadic decisions.

## Supplementary Information

Additional file 1.

## Data Availability

The dataset supporting the conclusions of this article and the associated analysis scripts are available at the first author’s Github repository: https://github.com/mattdblanchard/article_trait_conf_comms_dyads.

## Abbreviations

EAT: Esoteric analogies test
LMM: Linear mixed-effects model
RAPM: Raven’s advanced progressive matrices
