# Supplementary material for: How trait confidence and communication shape dyadic decision outcomes and confidence matching
Source: Cogn Res Princ Implic. 2026 Jan 31;11:10. doi: 10.1186/s41235-026-00705-1 (PMC12860768; doi:10.1186/s41235-026-00705-1)
Supplement: Supplementary file 1 — Additional file 1. [file 41235_2026_705_MOESM1_ESM.docx]

**Supplementary Materials**

**Appendix A**

**Development Of The General-Knowledge Tests**

Test development was conducted to construct 3 versions of a general knowledge test to administer in the main study. These versions were required to be matched on decision accuracy, decision confidence, and content domains.

**Participants**

Participants were 67 undergraduate psychology students (22 males, Mean age = 20.27, SD = 2.97) who completed the study for partial course credit. These participants were ineligible for participation in the later stages of this study.

**Measures**

The items for the general-knowledge test were collected from several previous studies (Brewer & Sampaio, 2012; Blanchard et al. 2020; Schuldt et al., 2017; Stankov, 1997) and constructed by members of the researcher team. 101 two alternative items were selected to cover a broad range of content areas: geography, art, music, film, history, science, and vocabulary. For example, *What does the word orthodox mean? Religious or Conventional** and *Who wrote the novel titled Brave New World? Aldous Huxley* or George Orwell* (* indicates the correct answer). After each item, participants were asked to provide a confidence rating ranging from 50% (guessing) to 100% (completely certain) for the correctness of their response. Of the 101 items, 22 were selected to form each of the 3 general-knowledge tests. By design, the three versions were matched on decision accuracy, decision confidence, and content domain.

**Procedure**

All participants completed the test development stage in a university computer lab. After providing consent, participants completed a demographic questionnaire then answered all general-knowledge items by themselves in a randomised order.

**Test Development**

Overall, mean decision accuracy was 64.49 (SD = 21.64) and mean decision confidence was 73.40 (SD = 20.41) for the general-knowledge items. We used item-level decision accuracy and decision confidence to construct 3 different versions of the general-knowledge test that had equivalent mean decision accuracy and decision confidence. Table A1 demonstrates the descriptive statistics for each version of the general-knowledge test that we constructed from the total pool of items. There were no significant differences on mean decision accuracy (*F* = .04, *p* = .84) or decision confidence (*F* = .01, *p* = .91) between the three matched versions of the general-knowledge tests. Omega total was used to measure internal consistency (McDonald, 1999). For decision accuracy, internal consistency was acceptable for exploratory research purposes for version 2 and 3 (ω_t_ = .56 and .63, respectively) but was low for version 1 (ω_t_ = .47). For decision confidence, internal consistency was good to excellent (ω_t_ ranging from .75 to .82).

Table A1

*Descriptive Statistics For The Matched Versions Of The General Knowledge Test (N = 67)*

|  | Version | | |  |
| --- | --- | --- | --- | --- |
|  | 1 | 2 | 3 | *F* |
| Decision Accuracy |  |  |  |  |
| Mean | 58.28 (0.20) | 58.07 (0.20) | 57.94 (.20) | 0.04 |
| ω_t_ | .47 | .56 | .63 |  |
| Decision Confidence |  |  |  |  |
| Mean | 71.64 (19.56) | 71.58 (20.29) | 71.70 (19.92) | 0.01 |
| ω_t_ | .81 | .75 | .82 |  |

Note. ω_t_ = Omega total.

**Appendix B**

**Pre-screening Study**

The pre-screening study was used to identify participants who were high-trait or low-trait confidence for inclusion in the main study. Our selection criteria aimed to recruit individuals who scored within ±1.50 standard deviations of the mean on cognitive ability and beyond ±0.50 standard deviations on trait confidence. The target sample size for the main study was 210 participants comprised of 105 high-trait and 105 low-trait confidence individuals who would be paired into 35 dyads in each of the trait confidence categories: low-trait, mixed-trait, and high-trait.

Figure B1 displays the distribution of standardised trait confidence and cognitive ability scores from the screening study and highlights the subset of participants who completed the main study. As shown, most selected individuals fall within the intended ranges on trait confidence and cognitive ability. However, due to attrition, a small number of participants fall slightly outside these thresholds. One notable pattern is that high-trait confidence participants in the main study tend to cluster towards the upper end of the cognitive ability target range more than low-trait confidence individuals. This imbalance necessitated statistical control for the effect of cognitive ability in the main study analyses. Figure B2 presents histograms for both variables of the distributions for participants recruited in the main study.

Figure B1

*Distribution Of Trait Confidence And Cognitive Ability Scores In The Screening Study With Highlighted Main Study Participants (N = 1189)*


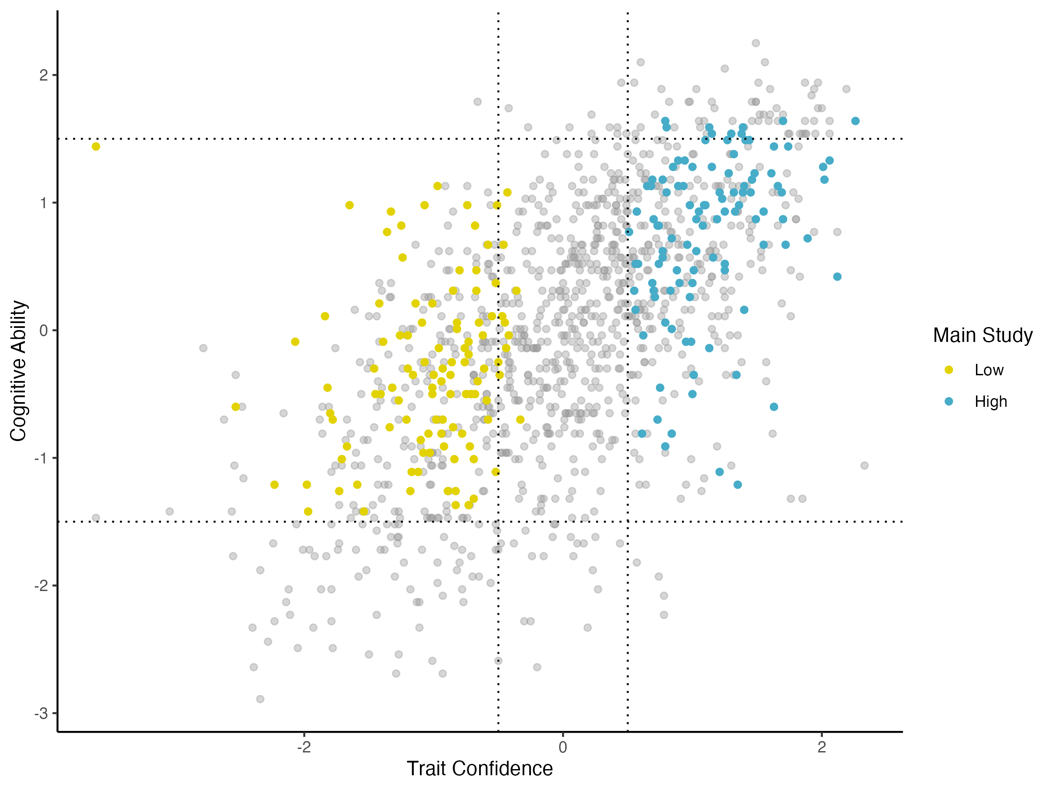


Figure B2

*Histograms Of Trait Confidence And Cognitive Ability Scores In The Main Study (N = 210)*

**
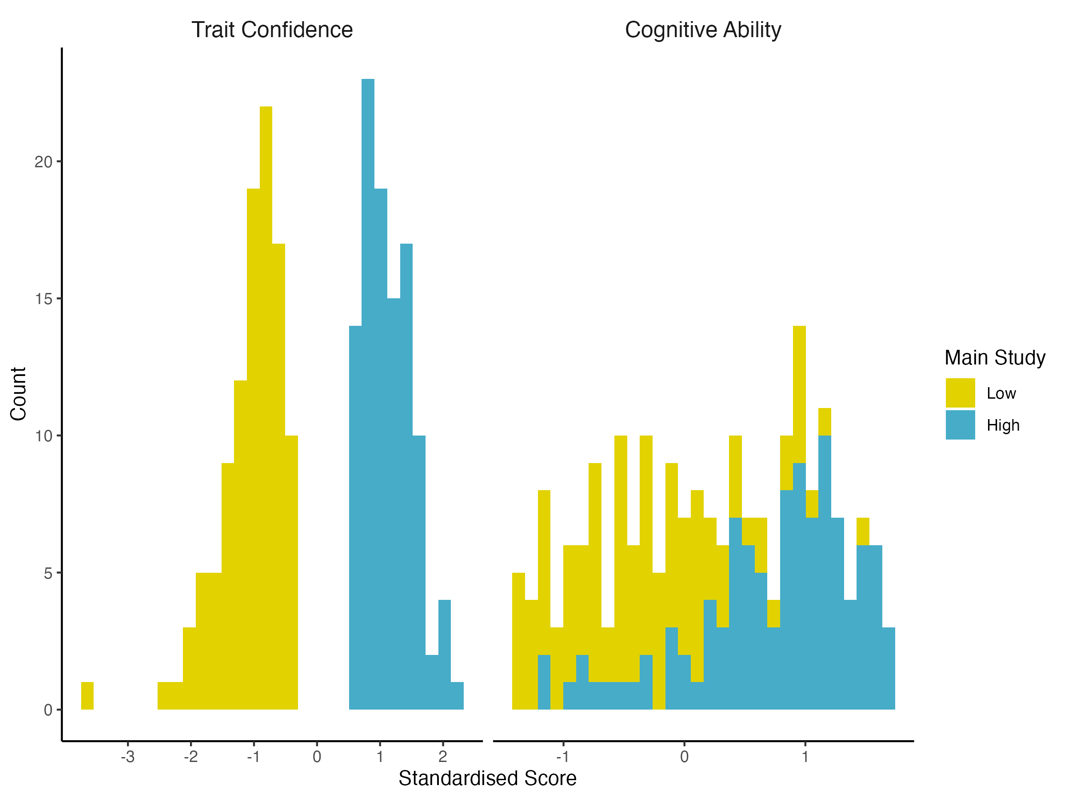
**

**Participants**

Participants were 1251 Australian university students (370 males; mean age = 20.89, SD = 5.01) who completed the study for either partial course credit or financial reimbursement. A total of 62 participants were excluded for non-genuine responses for Raven’s Advanced Progressive Matrices (RAPM) or Esoteric Analogies Test (EAT). The final sample were 1189 participants (343 males; mean age = 20.91, SD = 5.09) who were screened for trait confidence and cognitive ability.

## **Measures**

*Raven’s Advanced Progressive Matrices* (RAPM; Raven, 1938-65): This test consists of 36 items, each featuring a 3x3 grid of abstract figures forming a horizontal and vertical pattern, with the bottom right figure missing. Participants select one of eight possible options to complete the matrix. Accuracy is a measure of Fluid Reasoning. The internal consistency has been shown to be excellent for accuracy, ranging from .80 to .81, and decision confidence, ranging from .90 to .92 (Blanchard et al., 2020; Blanchard et al., 2023). After responding to each item, participants rated their decision confidence in their answer on a scale from 12.5% (guessing) to 100% (completely certain). In the present study, participants completed a short 15-item version.

*Mini-IPIP* (Donnellan et al., 2006): This questionnaire presented participants with 20 statements about their personality, which they rated on a five-point scale ranging from "very inaccurate" (1) to "very accurate" (5). For example, one item asked participants to rate the accuracy of the statement “Am the life of the party.” This scale assesses the Big Five personality traits and has been found to have acceptable internal consistency for Agreeableness (.70), Conscientiousness (.69), Extraversion (.77), Intellect (.65), and Neuroticism (.68).

*Esoteric Analogies Test* (EAT; Stankov, 1997): This measure involved participants completing 20 of the original 24 analogies. For each analogy, participants were presented with a pair of words and asked to select one of four options that reflected the same relationship with a target word. For example, *LOVE is to HATE as FRIEND is to: (1) LOVER, (2) PAL, (3) OBEY, (4) ENEMY*.* Accuracy requires both reasoning skills and prior knowledge thus it is a mixed measure of Fluid Reasoning and Crystallised Intelligence. Prior research with Australian undergraduate samples reported acceptable internal consistency for accuracy (ranging from .69 to .74) and excellent internal consistency for decision confidence (ranging from .88 to .94; Jackson et al., 2016; Law et al., 2022).

**Procedure**

All participants completed the 30-minute screening study remotely using their own device and internet connection. After providing consent, participants completed the tasks in the same order: demographic questionnaire, RAPM, mini-IPIP, and EAT. All measures were completed individually.

**Appendix C**

**The Items For The Final Versions Of The General Knowledge Tests Used In The Main Study**

Table C1

*The General Knowledge Test Items In Version 1 Shown in the Isolated Condition*

| *Item* | *Question* | *Option 1* | *Option 2* | *Correct Response* |
| --- | --- | --- | --- | --- |
| *1* | What does the word duress mean? | Period of time | Compulsion | Compulsion |
| *2* | What does the word abjure mean? | Renounce | Arrest | Renounce |
| *3* | Who wrote the novel titled Frankenstein? | Mary Shelley | Jane Austen | Mary Shelley |
| *4* | Who was the first president of the United States? | Abraham Lincoln | George Washington | George Washington |
| *5* | What does the word gush mean? | Spurt | Cry | Spurt |
| *6* | Approximately how many Australian Aboriginal languages are there? | 150 | 300 | 300 |
| *7* | The Dalai Lama is from which country? | Tibet | India | Tibet |
| *8* | In which city would you find the most famous works by Antoni Gaudi? | New York | Barcelona | Barcelona |
| *9* | Which school of art did the painter Claude Monet come from? | Impressionism | Expressionism | Impressionism |
| *10* | What does the word unwary mean? | Tireless | Incautious | Incautious |

Table C2

*The General Knowledge Test Items In Version Shown in the Passive Condition*

| *Item* | *Question* | *Option 1* | *Option 2* | *Correct Response* |
| --- | --- | --- | --- | --- |
| *1* | Which is a stress hormone produced by the human body? | Serotonin | Cortisol | Cortisol |
| *2* | Andy Warhol designed an album cover for which band? | The Beatles | The Rolling Stones | The Rolling Stones |
| *3* | How many feature films has Quentin Tarantino directed? | 7 | 9 | 9 |
| *4* | Who wrote the novel titled The Handmaid's Tale? | John Steinbeck | Margaret Atwood | Margaret Atwood |
| *5* | What does the word perspire mean? | Struggle | Sweat | Sweat |
| *6* | In which year did the Berlin wall fall? | 1989 | 1979 | 1989 |
| *7* | Which planet is larger in size? | Neptune | Jupiter | Jupiter |
| *8* | The Persistence of Memory is a painting by which artist? | Salvador Dali | Henri Matisse | Salvador Dali |
| *9* | What does the word feign mean? | Pretend | Be cautious | Pretend |
| *10* | A deficiency of vitamin C causes which disorder? | Scurvy | Rickets | Scurvy |

Table C3

*The General Knowledge Test Items In Version 3 Shown in the Active Condition*

| *Item* | *Question* | *Option 1* | *Option 2* | *Correct Response* |
| --- | --- | --- | --- | --- |
| *1* | Which continent has a larger area? | Antarctica | Europe | Antarctica |
| *2* | Which country was Pablo Picasso born in? | France | Spain | Spain |
| *3* | For which film did Cate Blanchett win an Oscar award for Best Actress? | Blue Jasmine | Carol | Blue Jasmine |
| *4* | Who wrote the novel titled Anna Karenina? | Ernest Hemmingway | Leo Tolstoy | Leo Tolstoy |
| *5* | When did Sydney host the Summer Olympic Games? | 2004 | 2000 | 2000 |
| *6* | What does the word dyschronometria mean? | Impaired ability to estimate amount of time passed | Impaired ability to maintain a line of thought | Impaired ability to estimate amount of time passed |
| *7* | Which continent has a larger area? | Europe | North America | North America |
| *8* | Who wrote the Australian novel titled Cloudstreet? | Tim Winton | Christos Tsiolkas | Tim Winton |
| *9* | What does the word orthodox mean? | Religious | Conventional | Conventional |
| *10* | Who painted the ceiling of the Sistine Chapel? | Michelangelo | Raphael | Michelangelo |

**Appendix D**

**Individual Differences Measures From The Main Study**

*Motivational Traits Scale* (Haesevoets et al., 2019): This scale consisted of 57 items that assessed 3 motivational traits associated with social decision-making: prosocial, proself, and fearful. The items were taken from scales that assess fairness (Van Hiel et al., 2008), altruism (Tazelaar et al., 2004), social welfare concerns (Haesevoets et al., 2018), concern for others (Selenta & Lord, 2005), greed (Krekels & Pandelaere, 2015), competitiveness (﻿Xie et al., 2006), entitlement (﻿Campbell et al., 2004), fear (﻿Van Hiel et al., 2008) and risk aversion (Mandrik & Bao, 2005). For example, participants rated their agreement with statements like, *When I have to make a decision that also influences others I want to make a decision that leads to an equal outcome for everyone,* using a 7-point Likert scale, ranging from *Strongly Disagree (1)* to *Strongly Agree (7)*. The underlying scales have demonstrated good internal consistency with Cronbach's Alpha ranging from of .74 to .93.

*Trust Scale* (McAllister, 1995; Wilson et al., 2006): This scale uses 5 items to measure trust between members of small groups. It is appropriate for computer-mediated communication and McAllister’s original scale was adapted by Wilson and colleagues for suitability with a student population. Participants rated their agreement with statements like *I can freely share my ideas and feelings in this group* using a 5-point Likert scale, ranging from *Strongly Disagree* (1) to *Strongly Agree* (5). Higher scores reflect greater trust withing a team. The scale has been shown to possess excellent internal consistency for cognitive trust and affective trust which have Cronbach’s Alpha values of .82 and .88 respectively.

*Psychological Safety Scale* (Edmondson, 1999): This scale uses 7 items to measure the extent to which individual team members feel safe to take interpersonal risks within their team. Participants rated their agreement with statements such as *If you make a mistake on this team, it is often held against you*, using a 5-point Likert scale from *Strongly Disagree* (1) to *Strongly Agree* (2). Higher scores reflect greater psychological safety within a team. The scale possess excellent internal consistency, with Cronbach’s alpha of .82 in the original study.

*Empathy Quotient* (Baron-Cohen & Wheelwright, 2004): The Empathy Quotient is a 60-item self-report questionnaire designed to measure empathy, which is the ability to understand and respond to the emotions and mental states of others. Of the 60 items, 40 assess empathy and 20 are control items. Participants responded to statements such as, *I can easily tell if someone is upset, even if they don’t say anything,* using a 4-point Likert scale ranging from *Strongly Disagree* (1) to *Strongly Agree* (4). Higher scores reflect greater empathy. The scale captures both cognitive empathy (understanding the mental states of others) and affective empathy (the capacity to respond emotionally to the feelings of others). Internal consistency has been shown to be excellent, with Cronbach’s alpha values around .92.

*Reading the Mind in the Eyes Test* (Baron-Cohen et al., 2001). This is a 36-item measure of emotion perception that assesses one’s ability to infer emotional states from images of people’s eyes. Woolley et al., (2010) used this measure to assess social sensitivity. Participants are presented with an image of someone’s eyes and must quickly select the word (from four options) that best describes the thought or feeling expressed by the person in the image. Higher scores indicate greater ability at perceiving the emotions of others. In the present study, participants completed a short 10-item version (Olderbak et al., 2015) which has been shown to possess good internal consistency, with Cronbach’s Alpha of .73.

*Behavioral Inhibition System/Behavioral Activation System Scales* (BIS/BAS; Carver & White, 1994): The BIS/BAS consists of 24 items designed to measure individual differences in sensitivity to punishment (BIS) and reward (BAS). The BIS subscale assesses the degree to which participants experience behavioural inhibition in response to potential punishment or negative outcomes. In contrast, the BAS subscale captures the extent to which individuals are driven by rewards, divided into three components: Reward Responsiveness, Drive, and Fun Seeking. Participants rated statements such as *Criticism or scolding hurts me quite a bit* on a four-point scale, from *Very true for me* (1) to *Very false for me* (4)*.* The internal consistency of the scales is good with Cronbach’s Alpha scores of .74 for the BIS, .73 for the BAS Reward Responsiveness, .76 for the Bas Drive, and .66 for the BAS Fun Seeking subscales.

*Risk Aversion* (Holt & Laury, 2002). This behavioural measure comprised 10 items where participants made choices between two lottery options. Option A offered smaller, more stable payouts (lower risk), while Option B provided larger, more variable payouts (higher risk). For instance, in the first item, Option A gave a 1/10 chance of paying $2.00 and a 9/10 chance of paying $1.60, while Option B had a 1/10 chance of paying $3.85 and a 9/10 chance of paying only $0.10. Participants were required to choose which lottery they would prefer. Across items, the payout amounts for both options remained constant, but the probabilities of winning shifted incrementally with each successive item, moving by 1/10 with each step. By the final item, the odds reached 10/10 for both options, fully favouring the prize with the higher payout. This gradually increased the likelihood of the larger payout. The point at which a participant switches from selecting Option A to Option B provides an indicator of their risk aversion. For example, choosing Option B on the first item (when the probability of the higher payout is just 1/10) indicates low risk aversion, whereas continuing to choose Option A on item 8 (where the probability of a high payout is 8/10) suggests very high risk aversion.

*Communication Measures.* Conversations between dyad members were recorded during the active communication condition. From these recordings, we calculated the number of speaking turns and the equality of turn-taking. Following Woolley et al. (2010), equality of turn-taking was measured by computing a standard deviation for the total number of speaking turns of a dyad’s members. A zero-value indicated perfect equality, where both members contributed an equal number of turns, while higher values reflected increasing levels of inequality. For clarity, we referred to this measure as *inequality of turn-taking*.

**Descriptive Statistics**

Table D1 displays the descriptive statistics and internal consistency estimates for the additional individual difference variables. Internal consistency estimates ranged from acceptable (.64) to excellent (.91) for all psychological measures except social sensitivity which was low (.47).

The *inequality of* communication variables represent the similarity between dyad members for the number of speaking turns (inequality of turn-taking) and the number of words spoken (inequality of words spoken). A score of zero on either of the inequality variables indicates that dyad members had an identical number of speaking turns or words spoken and higher values indicated greater inequality.

The *total* communication variables represent the total number of speaking turns (total talking turns) and the total number of words spoken (total words spoken) for both dyad members. Internal consistency estimates were acceptable for inequality of turn-taking (.59) and good for the other communication variables, ranging from .74 to .83.

Table D1

*Descriptive Statistics And Internal Consistency Estimates For Individual Differences Variables For Each Trait Confidence Condition* (*N* = 105)

|  |  | Trait Confidence | | |  |
| --- | --- | --- | --- | --- | --- |
|  |  | Low | Mixed | High |  |
| Variable | IC | Mean (SD) | Mean (SD) | Mean (SD) | *F_2, 102_* |
| Social Sensitivity | .47 | 76.19 (20.96) | 79.05 (17.88) | 72.38 (27.93) | 1.53 |
| EQ | .89 | 44.94 (12.35) | 43.51 (11.77) | 42.43 (11.55) | 0.79 |
| BIS Total | .82 | 22.50 (3.64) | 21.84 (4.49) | 21.53 (3.33) | 1.16 |
| BAS Drive | .75 | 10.74 (2.32) | 11.29 (2.11) | 10.80 (2.35) | 1.22 |
| BAS Fun | .70 | 12.03 (2.13) | 11.86 (2.35) | 12.24 (2.10) | 0.54 |
| BAS Reward | .70 | 17.43 (1.77) | 17.03 (2.31) | 17.20 (2.15) | 0.65 |
| Proself Factor | - | -0.01 (0.46) | 0.03 (0.44) | -0.02 (0.47) | 0.22 |
| Prosocial Factor | - | 0.08 (0.58) | -0.06 (0.55) | -0.03 (0.50) | 1.29 |
| Fearful Factor | - | 0.06 (0.50) | 0.00 (0.61) | -0.07 (0.56) | 0.97 |
| Risk Aversion | .76 | 4.87 (2.31) | 4.30 (2.06) | 4.50 (2.14) | 1.25 |
| Psychological Safety | .64 | 5.58 (0.79) | 5.74 (0.65) | 5.64 (0.67) | 0.88 |
| Trust | .72 | 4.16 (0.51) | 4.15 (0.50) | 4.05 (0.49) | 0.95 |
| Inequality Turn Taking | .59 | 2.48 (2.20) | 2.91 (3.71) | 2.45 (1.96) | 0.30 |
| Inequality Words Spoken | .74 | 123.10 (115.08) | 114.03 (97.64) | 92.80 (74.79) | 0.88 |
| Total Talking Turns | .81 | 109.40 (30.99) | 105.37 (26.32) | 110.53 (32.06) | 0.29 |
| Total Words Spoken | .83 | 880.14 (403.23) | 772.80 (292.11) | 835.24 (356.44) | 0.81 |

**Appendix E**

**Selecting The Best Fitting Model For Decision Accuracy And Decision Confidence**

We compared eight models to select the best fitting model to include in the main analyses for decision accuracy and decision confidence. All models included the same fixed effects structure that included three-way interactions for grouping (individual vs dyad), communication type (isolated vs passive vs active), and trait confidence (low-trait vs mixed-trait vs high-trait), along with covariates for two cognitive abilities (EAT accuracy and RAPM accuracy). The models differed only in their random effects structure.

For each outcome, the models compared were: 1) linear regression with fixed effects only (no random effects were included); 2) random intercepts for each individual; 3) random intercepts for each dyad; 4) random intercepts for both individuals and dyads; and 5) random intercepts and slopes for communication type across individuals; 6) random intercepts and slopes for communication type across dyads; 7) random intercepts and slopes for communication type across individuals and random intercepts for dyads; 8) random intercepts and slopes for communication type at both the individual and dyad levels. We compared the models on Akaike Information Criterion (AIC), Bayesian Information Criterion (BIC), and log-likelihood values. Likelihood ratio tests (LRTs) compared nested models relative to simpler alternatives, with statistical significance indicated where relevant. See Table E1 for the model comparisons.

Table E1

*Comparison Of The Models For Each Outcome*

|  | Decision Accuracy | | | Decision Confidence | | |
| --- | --- | --- | --- | --- | --- | --- |
| Model | AIC | BIC | LRT | AIC | BIC | LRT |
| 1 | 10542 | 10650 | -5250 | 9300 | 9408 | -4629 |
| 2 | 10328 | 10441 | -5142^***^ | 8470 | 8583 | -4213^***^ |
| 3 | 10358 | 10471 | -5157 | 8806 | 8919 | -4381 |
| 4 | 10312 | 10431 | -5133^***^ | 8456 | 8574 | -4205^***^ |
| 5 | 10128 | 10267 | -5037^***^ | 8058 | 8197 | -4002^***^ |
| 6 | 10290 | 10428 | -5118 | 8794 | 8933 | -4370 |
| 7 | 10107 | 10250 | -5025^***^ | 8035 | 8179 | -3990^***^ |
| 8 | 10079 | 10249 | -5007^***^ | 8023 | 8192 | -3978^***^ |

Model 1: no random effects

Model 2: (1 | individual)

Model 3: (1 | dyad)

Model 4: (1 | individual) + (1 | dyad)

Model 5: (1 + communication | individual)

Model 6: (1 + communication | dyad)

Model 7: (1 + communication | individual) + (1 | dyad)

Model 8: (1 + communication | individual) + (1 + communication | dyad)

^***^ *p* < .001.

For both outcomes, Model 8, which included random intercepts and slopes for communication at both the individual and dyad levels, provided the best fit based on AIC, BIC, and log-likelihood, with significant improvements over all simpler models. Despite increased model complexity, Model 8 did not exhibit convergence or singularity issues and was therefore retained as the final model for both decision accuracy and decision confidence analyses.

**Appendix F**

**Additional Results For The Final Models For Decision Accuracy And Decision Confidence**

Figure F1

*Mean Scores On Each Outcome For The Three Conditions*


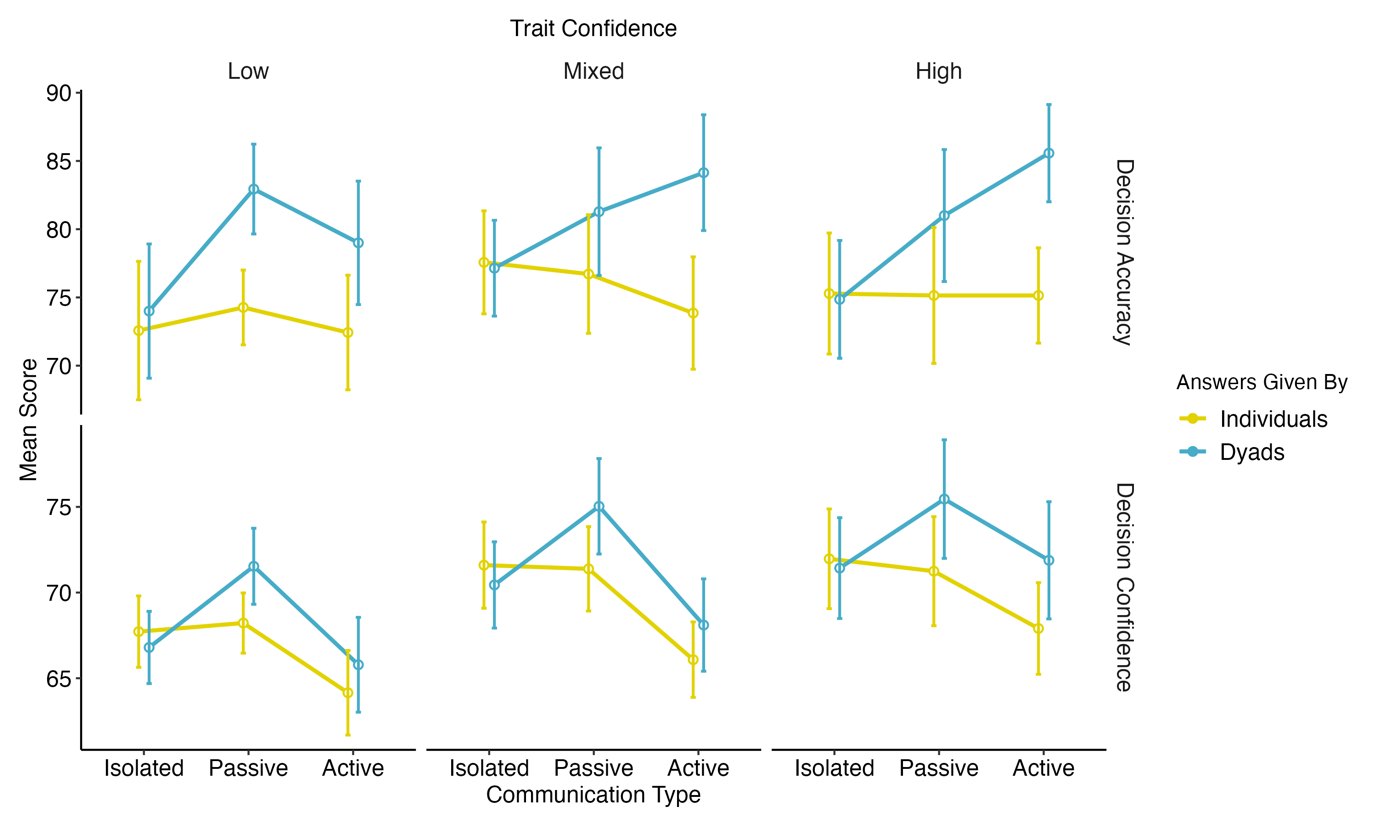


Table F1
*Random Effects For Communication Within Individuals and Dyads For Decision Accuracy And Decision Confidence*

|  | Individual | | | Dyad | | |
| --- | --- | --- | --- | --- | --- | --- |
| Outcome | Intercept  SD | Passive  SD | Active  SD | Intercept  SD | Passive  SD | Active  SD |
| Decision accuracy | 13.77 | 14.98 | 11.95 | 6.75 | 6.01 | 9.35 |
| Decision confidence | 9.02 | 7.14 | 6.47 | 3.82 | 3.42 | 2.91 |

Table F2

*Baseline Comparisons For Individual Responses*

|  | Decision | | | |
| --- | --- | --- | --- | --- |
|  | Accuracy | | Confidence | |
| Predictor | b | SE | *b* | SE |
| *Low* |  |  |  |  |
| Passive vs Isolated | 1.73 | 2.62 | 0.76 | 1.18 |
| Active vs Isolated | -0.14 | 2.50 | -3.57^**^ | 1.07 |
| Active vs Passive | -1.87 | 2.58 | -4.33^***^ | 1.13 |
| Mixed |  |  |  |  |
| Passive vs Isolated | -0.86 | 2.60 | -0.22 | 1.16 |
| Active vs Isolated | -3.71 | 2.50 | -5.52^***^ | 1.07 |
| Active vs Passive | -2.86 | 2.56 | -5.30^***^ | 1.12 |
| High |  |  |  |  |
| Passive vs Isolated | -0.14 | 2.60 | -0.72 | 1.16 |
| Active vs Isolated | -0.14 | 2.50 | -4.07^***^ | 1.07 |
| Active vs Passive | 0.00 | 2.56 | -3.34^**^ | 1.12 |

^***^ *p* < .001; ^**^ *p* < .01.

Table F3

*Difference Of The Differences For The Three-Way Interaction Effects*

|  |  |  | Decision | | | |
| --- | --- | --- | --- | --- | --- | --- |
|  |  |  | Accuracy | | Confidence | |
| *Trait Confidence* | *Grouping* | *Communication* | b | SE | *b* | SE |
| Low | Dyad vs Ind | Passive vs Isolated | 7.25^**^ | 2.25 | 4.23^***^ | 0.77 |
| Low | Dyad vs Ind | Active vs Isolated | 5.14^*^ | 2.24 | 2.56^***^ | 0.77 |
| Low | Dyad vs Ind | Active vs Passive | -2.11 | 2.25 | -1.68^*^ | 0.77 |
| Mixed | Dyad vs Ind | Passive vs Isolated | 5.00^*^ | 2.24 | 4.81^***^ | 0.77 |
| Mixed | Dyad vs Ind | Active vs Isolated | 10.71^***^ | 2.24 | 3.17^***^ | 0.77 |
| Mixed | Dyad vs Ind | Active vs Passive | 5.71^*^ | 2.24 | -1.63^*^ | 0.77 |
| High | Dyad vs Ind | Passive vs Isolated | 6.29^**^ | 2.24 | 4.75^***^ | 0.77 |
| High | Dyad vs Ind | Active vs Isolated | 10.86^***^ | 2.24 | 4.52^***^ | 0.77 |
| High | Dyad vs Ind | Active vs Passive | 4.57^*^ | 2.24 | -0.23 | 0.77 |
| *Communication* | *Grouping* | *Trait Confidence* |  |  |  |  |
| Isolated | Dyad vs Ind | Low vs Mixed | -1.86 | 2.24 | -0.23 | 0.77 |
| Isolated | Dyad vs Ind | Low vs High | -1.86 | 2.24 | 0.38 | 0.77 |
| Isolated | Dyad vs Ind | Mixed vs High | 0.00 | 2.24 | 0.61 | 0.77 |
| Passive | Dyad vs Ind | Low vs Mixed | -4.11^†^ | 2.25 | 0.34 | 0.77 |
| Passive | Dyad vs Ind | Low vs High | -2.82 | 2.25 | 0.90 | 0.77 |
| Passive | Dyad vs Ind | Mixed vs High | 1.29 | 2.24 | 0.56 | 0.77 |
| Active | Dyad vs Ind | Low vs Mixed | 3.71^†^ | 2.24 | 0.38 | 0.77 |
| Active | Dyad vs Ind | Low vs High | 3.86^†^ | 2.24 | 2.34^**^ | 0.77 |
| Active | Dyad vs Ind | Mixed vs High | 0.14 | 2.24 | 1.96^*^ | 0.77 |

^***^ *p* < .001; ^**^ *p* < .01; ^*^ *p* < .05; ^†^ *p* < .10.

**Appendix G**

**Selecting The Best Fitting Model For Testing The Emergence Of Decision-Specific Confidence Matching**

**Random Effect Structure**

We compared three models to select the best fitting model to include in the main analyses for predicting decision-specific confidence matching at the dyad- and item-levels. All models included the same fixed effects structure which included the interaction between communication type (isolated vs passive vs active) and trait confidence (low-trait vs mixed-trait vs high-trait), along with covariates for the two cognitive abilities (EAT accuracy and RAPM accuracy). The models differed only in their random effects structure. Decision-specific confidence matching was operationalised as the initial confidence difference between members of a dyad before interaction (individual ratings) minus the confidence difference after interaction for each item (dyadic ratings). A positive value indicated more similar confidence ratings (greater confidence matching), a negative value indicated less similar confidence matching, and a score of zero indicated no change after interaction.

The following models were compared: 1) linear regression with no random effects; 2) random intercepts for each dyad and item; 3) random intercepts and correlated slopes for communication by dyad plus a random intercept for item. We compared the models on AIC, BIC, and log-likelihood values. Likelihood ratio tests (LRTs) compared nested models relative to simpler alternatives, with statistical significance indicated where relevant. See Table E1 for the model comparisons.

Table G1

*Comparison Of Models With Different Random Effects*

| Model | AIC | BIC | LRT |
| --- | --- | --- | --- |
| 1 | 24794 | 24866 | -12385 |
| 2 | 24740 | 24825 | -12356^***^ |
| 3 | - | - | - |

Model 1: no random effects

Model 2: (1 | dyad) + (1 | item number)

Model 3: (1 + communication | dyad) + (1 | item number)

^***^ *p* < .001.

Model 2, which included random intercepts for dyad and item number, provided the best fit based on AIC, BIC, and log-likelihood, with significant improvements over model 1. Model 3 was singular. Therefore, we retained model 2 as the final model.

**Interaction effect structure**

We compared 2 models to select the best fitting model for the main analyses. Each model had the same fixed effects as described above for model comparison of random effects. The models differed in their interaction effect structure. The models compared were: 1) main effects only (no interaction effects included); and 2) the addition of the two-way interaction effect. The results are presented in Table G2. Model 1 which had main effects only had the best fit, thus it was reported in the main text.

Table G2

*Comparison Of Models With Different Interaction Effect Structures*

| Model | AIC | BIC | LRT |
| --- | --- | --- | --- |
| 1 | 24736 | 24796 | - 12358 |
| 2 | 24740 | 24825 | - 12356 |

Model 1: main effects only
Model 2: two-way interaction effect added

**Appendix H**

**Full Model Results For Testing The Emergence Of Decision-Specific Confidence Matching**

The results for the final decision-specific confidence matching model are presented in Tables H1 which displays the main effects for each condition compared to no confidence matching (zero) and H2 which displays contrasts comparing confidence matching between conditions.

The model included random intercepts for both **dyad** and **item number**. The variance components indicated that **dyad-level differences** (**σ² = 2.85, SD = 1.69**) and **item-level differences** (**σ² = 5.17, SD = 2.27**) contributed **meaningful variation** in decision confidence changes. The **residual variance** was **σ² = 151.62, SD = 12.31**.

**Table H1**

*Main Effects For Decision-Specific Confidence Matching*

| *Variable* | Mean | SE | *t* |
| --- | --- | --- | --- |
| Isolated | 0.34 | 0.83 | 0.41 |
| Passive | 6.78 | 0.83 | 8.16^***^ |
| Active | 5.80 | 0.83 | 6.98^***^ |
| Low | 4.31 | 0.70 | 6.12^***^ |
| Mixed | 3.90 | 0.63 | 6.15^***^ |
| High | 4.70 | 0.73 | 6.42^***^ |
| RAPM Acc | 0.00 | 0.03 | -0.08 |
| EAT Acc | -0.01 | 0.03 | -0.42 |

^***^ *p* < .001.

**Table H2**

*Contrasts For The Differences Between Conditions*

| *Contrast* | *b* | SE | *t* |
| --- | --- | --- | --- |
| Passive vs Isolated | -6.44 | 1.15 | -5.59^***^ |
| Active vs Isolated | -5.46 | 1.15 | -4.74^***^ |
| Active vs Passive | 0.98 | 1.15 | 0.85 |
| Mixed vs Low | 0.41 | 0.72 | 0.57 |
| High vs Low | -0.38 | 0.96 | -0.40 |
| High vs Mixed | -0.80 | 0.80 | -1.00 |

^***^ *p* < .001

**Appendix I**

**Selecting The Best Fitting Model For Decision-Specific Confidence Matching Predicting The Change In Decision Accuracy**

**Random Effect Structure**

We compared 5 models to select the best fitting model to include in the main analyses predicting the change in decision accuracy. Each model had the same fixed effects: decision-specific confidence matching difference score, communication type (isolated vs passive vs active), and trait confidence (low-trait vs mixed-trait vs high-trait). The models also included RAPM accuracy and EAT accuracy as covariates to control for differences in ability. The models differed in their random effects structure. See Table I1 for the model comparisons.

Table I1

*Comparison Of Models With Different Random Effects*

|  | Decision accuracy Change | | |
| --- | --- | --- | --- |
| Model | AIC | BIC | LRT |
| 1 | 2227 | 2306 | -1092 |
| 2 | 2229 | 2311 | -1092 |
| 3 | - | - | - |
| 4 | - | - | - |
| 5 | - | - | - |

Model 1: no random effects

Model 2: (1 | dyad)

Model 3: (1 | communication)

Model 4: (1 + communication) + (1 | dyad)

Model 5: (1 + communication | dyad)

Models 3 and 4 were singular and model 5 did not have enough data to support this structure. Model 2 with random intercepts for dyads did not significantly improve the fit above a linear regression with fixed effects only. Inspection of the random effects showed no variance across dyads (**σ² = 0.00, SD = 0.00**). Thus, model 1 was selected as the final model reported in the main results.

**Interaction effect structure**

We compared 3 models to select the best fitting model for the main analyses. Each model had the same variables included as fixed effects as the random effects analysis described in the prior section. The models differed only in the complexity of the interaction structures. Specifically, the models included: 1) main effects only (no interaction effects included); 2) the addition of all two-way interactions effects; and 3) the addition of three-way interaction effects. The results are presented in Table I2.

Table I2

*Comparison Of Models With Different Interaction Effect Structures*

|  | Decision accuracy Change | | |
| --- | --- | --- | --- |
| Model | AIC | BIC | *F* |
| 1 | 2234 | 2268 | -1108 |
| 2 | 2231 | 2294 | -1098^*^ |
| 3 | 2227 | 2306 | -1092^*^ |

Model 1: main effects only
Model 2: two-way interaction effects added
Model 3: three-way interaction effects added

Model 3 which included three-way interaction effects showed a significant improvement in model fit compared to model 2. Thus, we reported model 3 in the main analyses.

**Appendix J**

**Full Model Results For The Relationship Between Decision-Specific Confidence Matching And The Change In Decision Accuracy**

The results for the final confidence matching model predicting accuracy gains are presented across Table J1 through to Table J4. The distribution plots and correlations between decision-specific confidence matching and the change in accuracy are displayed in Figure J1. A positive relationship indicates that greater confidence alignment predicted greater accuracy gains for dyadic responses (post-interaction).
**Figure J1**

*The Distribution of Scores for Decision-Specific Confidence Matching and the Pre-Post Interaction Change in Accuracy*


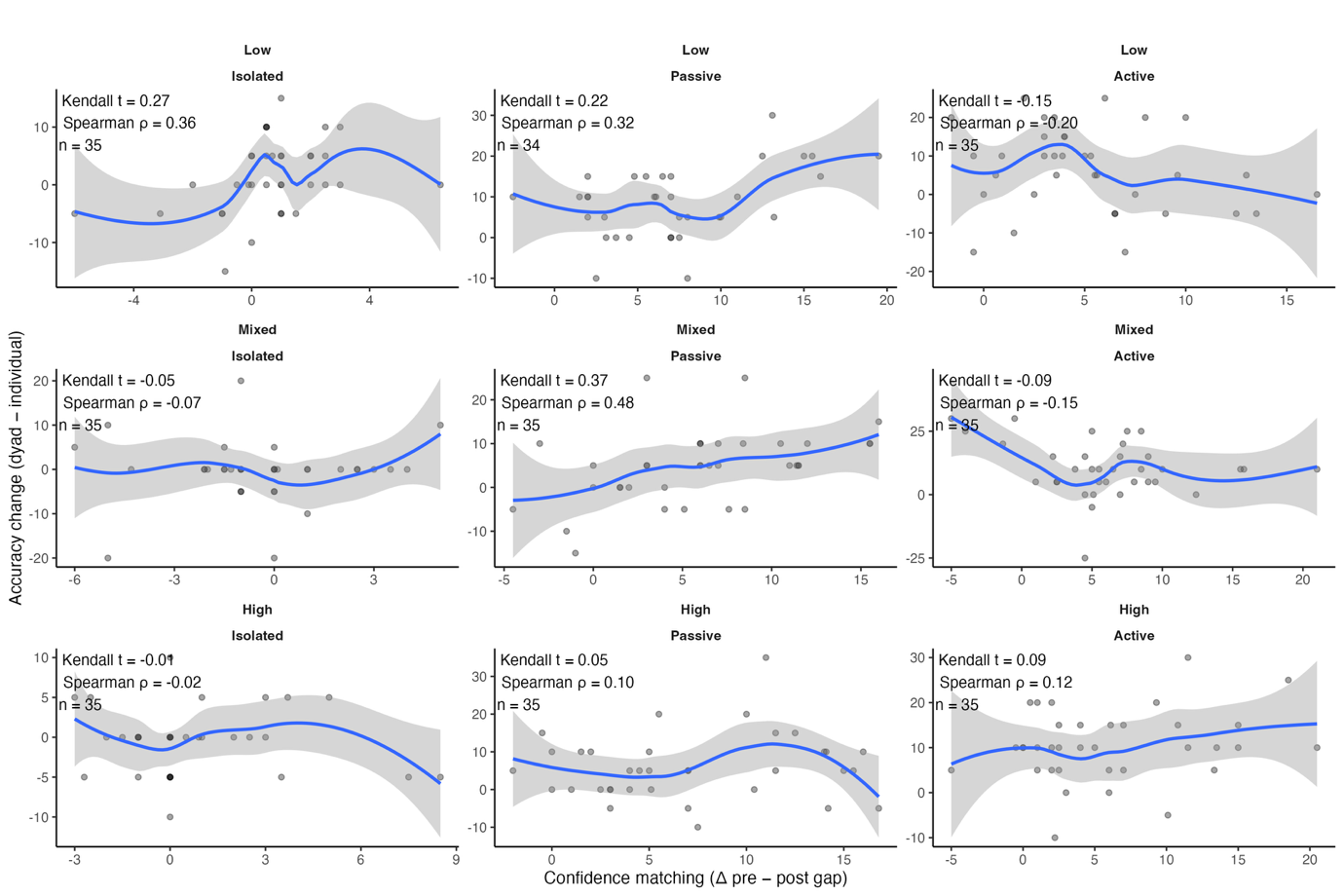


**Table J1**

*Omnibus Test Results*

| Term | *df* | *F* |
| --- | --- | --- |
| Intercept | 1,294 | 0.05 |
| Confidence matching | 1,294 | 2.28 |
| Communication | 2,294 | 5.55^**^ |
| Confidence | 2,294 | 0.09 |
| RAPM accuracy (centered) | 1,294 | 0.01 |
| EAT accuracy (centered) | 1,294 | 3.03^†^ |
| Confidence matching x Communication | 2,294 | 5.27^**^ |
| Confidence matching x Trait confidence | 2,294 | 0.90 |
| Communication x Trait confidence | 4,294 | 1.25 |
| Confidence matching x Communication x Trait confidence | 4,294 | 2.79^*^ |

^**^ *p* < .01; ^*^ *p* < .05; ^†^ *p* < .10.

**Table J2**

*The Effect of Decision-Specific Confidence Matching in Each Condition Compared to No Effect*

| *Variable* | Mean | SE | *t* |
| --- | --- | --- | --- |
| Isolated | 0.34 | 0.83 | 0.41 |
| Passive | 6.78 | 0.83 | 8.16^***^ |
| Active | 5.80 | 0.83 | 6.98^***^ |
| Low | 4.31 | 0.70 | 6.12^***^ |
| Mixed | 3.90 | 0.63 | 6.15^***^ |
| High | 4.70 | 0.73 | 6.42^***^ |
| RAPM Acc | 0.00 | 0.03 | -0.08 |
| EAT Acc | -0.01 | 0.03 | -0.42 |

^***^ *p* < .001.

**Table J3**

*Three-way Interactions for the Decision-Specific Confidence Matching Slope Predicting Accuracy Gains*

| Communication | Trait Confidence | Confidence Matching Slope | SE | *t*_294_ |
| --- | --- | --- | --- | --- |
| Isolated | Low | 1.04 | 0.69 | 1.51 |
| Isolated | Mixed | 0.21 | 0.56 | 0.37 |
| Isolated | High | -0.13 | 0.54 | -0.23 |
| Passive | Low | 0.75 | 0.28 | 2.64^**^ |
| Passive | Mixed | 0.70 | 0.27 | 2.65^**^ |
| Passive | High | 0.15 | 0.26 | 0.59 |
| Active | Low | -0.55 | 0.32 | -1.70^†^ |
| Active | Mixed | -0.48 | 0.27 | -1.80^†^ |
| Active | High | 0.27 | 0.23 | 1.17 |

^**^ *p* < .01; ^†^ *p* < .10.

**Table J4**

*Three-way Interaction Pairwise Contrasts*

| Communication | Trait Confidence | Confidence Matching Difference | SE | *t*_294_ |
| --- | --- | --- | --- | --- |
| Isolated - Passive | Low | 0.29 | 0.75 | 0.39 |
| Isolated - Active | Low | 1.60 | 0.76 | 2.09^†^ |
| Passive - Active | Low | 1.31 | 0.43 | 3.02^**^ |
| Isolated - Passive | Mixed | -0.50 | 0.62 | -0.80 |
| Isolated - Active | Mixed | 0.69 | 0.62 | 1.11 |
| Passive - Active | Mixed | 1.19 | 0.38 | 3.14^**^ |
| Isolated - Passive | High | -0.28 | 0.60 | -0.47 |
| Isolated - Active | High | -0.40 | 0.59 | -0.68 |
| Passive - Active | High | -0.12 | 0.35 | -0.34 |
| Low - Mixed | Isolated | 0.84 | 0.89 | 0.94 |
| Low - High | Isolated | 1.17 | 0.88 | 1.33 |
| Mixed - High | Isolated | 0.33 | 0.78 | 0.43 |
| Low - Mixed | Passive | 0.05 | 0.39 | 0.13 |
| Low - High | Passive | 0.60 | 0.38 | 1.56 |
| Mixed - High | Passive | 0.55 | 0.37 | 1.49 |
| Low - Mixed | Active | -0.07 | 0.42 | -0.16 |
| Low - High | Active | -0.83 | 0.40 | -2.07 |
| Mixed - High | Active | -0.76 | 0.36 | -2.12 |

^**^ *p* < .01; ^†^ *p* < .10.
